# Maternal Cadmium Exposure Impairs Lactational Performance and Milk Quality in Mice

**DOI:** 10.3390/biology15100754

**Published:** 2026-05-09

**Authors:** Meiqian Kuang, Haigang Xu, Yujian Chen, Ziyang Lai, Caifang Ren, Pan Huang, Hongjun Huang

**Affiliations:** 1 School of Medicine, Jiangsu University, Zhenjiang 212013, China; 2212413120@stmail.ujs.edu.cn (H.X.); 3231504011@stmail.ujs.edu.cn (Y.C.); 3221401373@stmail.ujs.edu.cn (Z.L.); rcf@ujs.edu.cn (C.R.); phuang@ujs.edu.cn (P.H.); 2 The First Clinical Medical College, Hainan Medical University, Haikou 570100, China

**Keywords:** Cd, mammary, lactational performance, hormonal imbalance

## Abstract

Cadmium (Cd) is a ubiquitous heavy metal environmental toxicant, and exposure poses a persistent public health concern due to its bioaccumulative properties. However, the impact of maternal Cd exposure on mammary gland function during lactation remains insufficiently understood. In this study, we investigated the effects of Cd exposure during pregnancy and lactation on mammary gland development, hormonal homeostasis, and lactational performance using a drinking water exposure model. Pregnant 7-week-old ICR mice were randomly assigned into Control and Cd groups. The Control group received standard drinking water, while the Cd group was administered water supplemented with Cd (12 mg/L). Results demonstrated that maternal Cd exposure markedly reduced maternal and offspring weight, respectively. Histological analysis revealed that Cd exposure resulted in decreased numbers of mammary alveoli and widened interstitial spaces, indicating impaired mammary development. In addition, Cd exposure markedly compromised lactational performance, as reflected by reduced milk yield and decreased levels of milk protein, fat, and lactose across multiple lactation stages. Integrated metabolomic and transcriptomic analyses further revealed substantial alterations in hormonal profiles and metabolic pathways associated with mammary gland function. Notably, Cd accumulation was detected in mammary tissue and milk, accompanied by transcriptional changes in genes involved in milk synthesis and lipid metabolism. In conclusion, these findings indicated that maternal Cd exposure during pregnancy and lactation is associated with structural and functional alterations of the mammary gland, leading to reduced lactational performance and impaired offspring growth. The findings contribute to a better understanding of the risks posed by environmental Cd exposure, emphasizing the need for effective public health strategies to mitigate its impact on maternal and infant health.

## Simple Summary

Cadmium (Cd) is a pervasive environmental toxicant that may threaten maternal mammary gland function during pregnancy and lactation. Here, we investigated how maternal Cd exposure affects mammary gland function and lactation in mice. Pregnant mice exposed to Cd during gestation and lactation showed disrupted mammary gland structure, reduced milk yield, and decreased milk protein, fat, and lactose levels. Molecular analyses revealed suppression of lactation-related genes and altered hormonal signaling. Cd accumulation in milk was associated with impaired offspring growth. These findings highlight the detrimental impact of maternal Cd exposure on lactation and early offspring development.

## 1. Introduction

Cadmium (Cd) is a toxic heavy metal that is widely distributed in the environment, primarily through industrial emissions, agricultural practices, and the contamination of soil and water sources [1,2]. As environmental pollution intensifies globally, the potential risks posed by Cd, especially through water contamination, have become an increasingly urgent public health concern [3]. An increasing number of recent studies indicate that Cd, as an endocrine disruptor, can alter hormone metabolism and impair or alter the normal development and secretory function, compromising the health of pregnant women [4,5,6]. Further investigations revealed that excessive Cd accumulation in pregnant women can activate apoptosis and autophagy in human placental trophoblasts, reduce estrogen (E_2_) and progesterone secretion, inhibit placental vascular formation, and disrupt placental structural integrity [7]. Experimental studies have demonstrated that high-dose Cd exposure suppresses serum E_2_ and progesterone levels, while low-dose exposure induces hormonal imbalance manifested by an abnormal E_2_ to progesterone ratio in mice [8,9]. These findings suggest that gestational Cd exposure may substantially perturb maternal endocrine regulation, potentially disrupting hormonally driven metabolic adaptations critical for pregnancy maintenance.

The mammary gland is a vital endocrine and lactational organ in females, playing a critical role in maintaining maternal and infant health [10,11,12]. Its normal development and function rely on the precise regulation of multiple hormones, including E_2_, progesterone, prolactin (PRL), and follicle-stimulating hormone (FSH) [13]. However, the effect of Cd exposure on mammary metabolic regulation, lactational performance, and hormonal homeostasis during pregnancy is still unknown. Drinking water represents one of the major sources of Cd exposure in humans [14]. A drinking water-based Cd poisoning model not only simulates the common real-life scenario of long-term, high-level exposure but also provides a reliable experimental basis for investigating the effects of Cd on mammary gland function during pregnancy [14]. The present study was designed to establish a pregnancy mouse model of low-dose Cd exposure via drinking water, in order to systematically evaluate the effects of Cd on mammary gland development and lactational function. Furthermore, integrated metabolomic and transcriptomic approaches were applied to explore the potential molecular mechanisms involved. In our study, we aim to elucidate the adverse effects of Cd as an environmental endocrine disruptor on mammary gland function, further clarify its potential risks to maternal and infant health, and provide scientific evidence to support environmental health risk assessment, public health, and clinical interventions.

## 2. Materials and Methods

### 2.1. Construction of Networks

To investigate the potential mechanism by which Cd affects lactation function, we conducted a network toxicology analysis. Initially, we retrieved gene targets related to Cd exposure from the Comparative Toxicogenomics Database (CTD, https://ctdbase.org), a comprehensive platform that aggregates biological effect data for chemicals, metals, and other substances. In this study, we used “Cd” as the keyword to screen genes related to Cd in CTD. In addition, we used “Delayed Onset of Lactogenesis” as the keyword to search for genes related to lactation in the GeneCards Database (https://www.genecards.org/). GeneCards is a comprehensive gene database that compiles the genes related to various human diseases. By intersecting the genes identified in both the CTD and GeneCards datasets, we ultimately selected key genes for further analysis. Subsequently, we utilized Cytoscape software (3.10.4) to construct a “Cd—gene—Delayed Onset of Lactogenesis” network diagram, visualizing the relationships between Cd exposure genes and delayed onset of lactogenesis. Then, we conducted Kyoto Encyclopedia of Genes and Genomes (KEGG) enrichment analysis and Gene Ontology (GO) enrichment analysis (encompassing biological processes, cellular components, and molecular functions) on Metascape (https://metascape.org) for genes related to Cd and genes related to Delayed Onset of Lactogenesis, respectively. GO and KEGG enrichment analysis highlighted significant pathways associated with the intersected genes, enhancing our understanding of their potential mechanisms in Delayed Onset of Lactogenesis. Subsequently, we plotted the “Cd-Pathway-Delayed Onset of Lactogenesis” visual interaction network for the top 20 common pathways with the strongest correlation. Finally, a protein–protein interaction (PPI) network for visualizing cross-genes was constructed using the STRING database (https://cn.string-db.org) and visualizing the network based on maximal clique centrality (cytoHubba-impl-0.1) [15,16].

### 2.2. Animal Experimental Design

**Figure 1:**
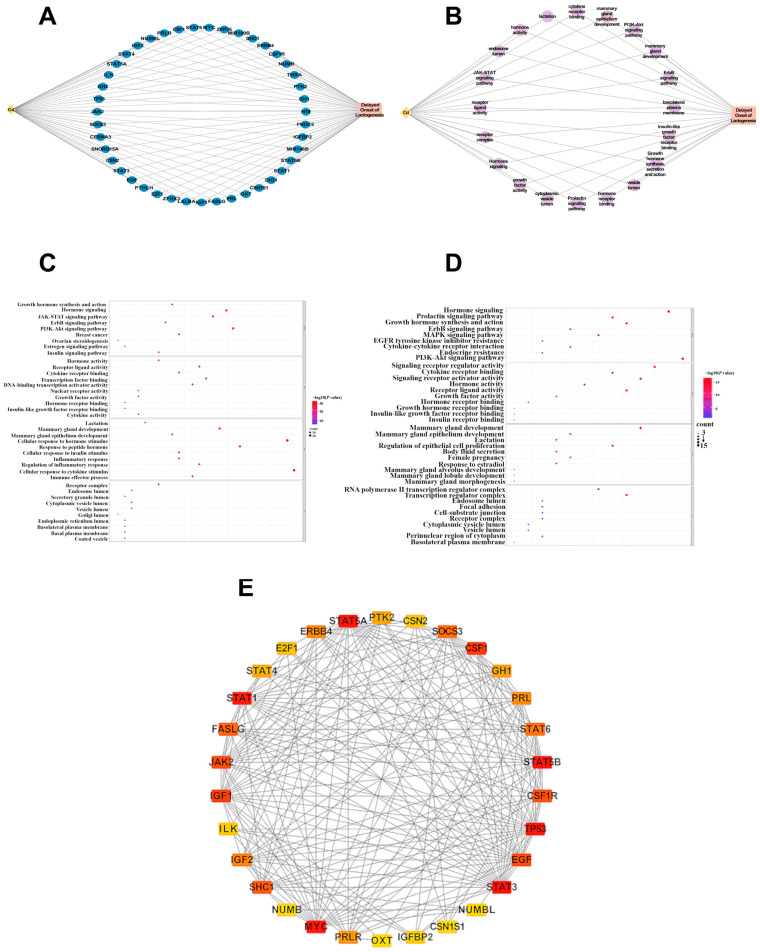
Network toxicology for Cd and delayed onset of lactogenesis. (**A**) Intersecting genes between Cd and Delayed Onset of Lactogenesis. (**B**) Intersecting pathways between Cd and Delayed Onset of Lactogenesis. (**C**) GO and KEGG enrichment analysis of Cd-related genes. (**D**) GO and KEGG enrichment analysis of delayed onset of lactogenesis-related genes. (**E**) The PPI network for intersecting genes. Greater importance is represented by a darker color.

All experiments involving mice were performed in accordance with the ethical policies and procedures approved by the Institutional Animal Care and Use Committee (IACUC) of Jiangsu University (approval number: 11920). This study was conducted in accordance with ARRIVE guidelines. A total of 24 7-week-old female ICR mice were obtained from the Animal Experiment Center of Jiangsu University and acclimatized for 1 week prior to experimentation. The female mice were paired with male mice at a ratio of 2:1, and the presence of a vaginal plug was designated as day 0 of pregnancy (PD 0). The mice were randomly allocated into two groups: a Control group (*n* = 12) and a Cd group (*n* = 12). The Control group dams received standard drinking water throughout the experiment. Cd group dams were administered normal water during early gestation (PD 0 to PD 5) and subsequently exposed to Cd-supplemented water (12 mg Cd/L) from PD 5 until lactation day 13 (LD 13). This exposure protocol spanned critical developmental phases, including late gestation (PD 5 to parturition), early lactation period (LD 0–LD 4), transition period (LD 4-LD 8), and mid-lactation period (LD 8–LD 13), see Figure 1A. Animals were housed under standardized conditions with a 12 h light/dark cycle, ambient temperature (22 ± 1 °C), and ad libitum access to food and water. At the specified time, following euthanasia on LD 14 via cervical dislocation under anesthesia, mammary gland tissues and whole blood samples were systematically collected. Mammary gland tissues were dissected, weighed, and processed for further analyses. Serum was isolated from whole blood via centrifugation and stored at −80 °C. Tissue specimens were either snap-frozen in liquid nitrogen for molecular analyses or fixed in 4% paraformaldehyde for histological examination.

### 2.3. Physiological Indicators Measurement

Maternal body weight was monitored using a calibrated electronic balance. During the gestational period (PD 0 to parturition), body weight was measured every 5 days. Postpartum, during the lactational phase, measurements were conducted every 4 days. Body weight changes were calculated as the percentage difference relative to PD 0 and interphase comparisons. Food intake and water consumption were recorded at 4-day intervals from PD 0 through LD 13. Cumulative datasets were compiled to calculate total gestational and lactational intake profiles.

### 2.4. Hematoxylin and Eosin (HE) Staining

The fourth mammary gland tissue was collected immediately after sacrifice and fixed in 4% paraformaldehyde at 4 °C for 48 h. After fixation, mammary gland tissue was dehydrated through graded ethanol, cleared in xylene, and embedded in paraffin. The 5 µm thickness samples were deparaffinized, graded ethanol (100%, 90%, 80%, and 70% ethanol), rehydrated, and sequentially stained with hematoxylin and eosin. Following mounting, stained mammary gland slides were observed and captured under a light microscope. Histological evaluation was performed qualitatively to assess overall mammary gland architecture, including alveolar density, epithelial organization, and interstitial space. Image assessment was conducted by two investigators in a blinded manner with respect to the experimental groups.

### 2.5. Lactation Performance Assessment

#### 2.5.1. Milk Yield Measurement

Maternal milk production was quantified using a validated pup-weight-based method at LD 13. To minimize variability in suckling stimulus, litter size was standardized to six pups per dam throughout the lactation period. Each dam nursed her own litter, and cross-fostering was not performed. Briefly, pups were separated from dams for 4 h (09:00–13:00) with pre-isolation litter weight (*W*1) recorded using a precision balance (±0.01 g). Post-isolation weight (*W*2) was measured before 1 h re-latching, followed by post-nursing weight (*W*3) determination. Hourly milk output was calculated as milk production (g/h) = (*W*3 − *W*2) + (*W*1 − *W*2)/4. Where (*W*1 − *W*2)/4 represents the metabolic weight loss correction during separation [17]. The same separation and re-latching procedures were applied consistently across all experimental groups to minimize potential variability associated with maternal behavior or stress.

#### 2.5.2. Milk Protein Quantification

Protein concentration in mammary secretions was quantified using the Bicinchoninic Acid (BCA) Assay (AL006, Nanjing ACE Biotechnology Co., Ltd., Nanjing, China). Milk samples from LD4, LD8, and LD12 were centrifuged to remove debris, and the supernatants were diluted with Phosphate Buffered Saline. A Bovine serum albumin standard curve (R^2^ > 0.99) was used for calibration. The diluted samples were mixed with the BCA working reagent, incubated at 37 °C for 30 min, and the absorbance was measured at 562 nm [18]. All measurements were performed in triplicate.

#### 2.5.3. Milk Triglyceride Content Analysis

Milk triglyceride (TG) concentrations were measured using a glycerol-3-phosphate oxidase-peroxidase enzymatic assay kit (A110-1-1, Nanjing Jiancheng Bioengineering Institute, Nanjing, China) according to the manufacturer’s instructions. After centrifugation, the milk samples from LD4, LD8, and LD12 were diluted and mixed with the working reagent. Following incubation at 37 °C for 10 min, the absorbance was measured at 500 nm [19,20]. A standard curve was used for quantification, and all samples were analyzed in triplicate. All measurements were performed in triplicate with blank controls.

### 2.6. Hormone ELISA Assay

Serum levels of FSH, progesterone, PRL, and E_2_ were quantified using enzyme-linked immunosorbent assay (ELISA) kits from BOYAN BIOTECH (Nanjing, China), with the following catalog numbers: FSH (No. BY-EM220239), progesterone (No. BY-JZF0066), PRL (No. BY-EM220246), and E_2_ (No. BY-JZF0048) [21,22]. Briefly, serum samples were centrifuged to remove particulates. Standards and diluted serum samples were loaded into pre-coated microplates in duplicate. After incubation with specific antibodies and subsequent washes, horseradish peroxidase (HRP)-conjugated detection antibodies were added. Following additional incubation and washing steps, tetramethylbenzidine (TMB) substrate was added for color development. The reaction was terminated with stop solution, and absorbance was measured at 450 nm using a microplate reader. Concentrations of each hormone were calculated based on standard curves generated from calibrators provided in the kits. All procedures were performed under strict adherence to the protocols outlined in the respective kit manuals.

### 2.7. Detection of Cd Concentration in Serum

Serum Cd concentrations were analyzed by Shanghai Preferred Biotechnology Co., Ltd. (Shanghai, China). Whole blood samples were centrifuged at 15,000× *g* for 10 min at 4 °C to separate serum. The resulting serum was further centrifuged at 3000× *g* for 15 min at 4 °C to remove residual cellular debris. Supernatants were aliquoted into pre-labeled cryovials, snap-frozen in liquid nitrogen, and transported to the testing facility under cryogenic conditions to preserve analyte stability.

### 2.8. Untargeted Metabolomic Analysis

Mouse mammary gland tissue samples were collected and submitted to Shanghai Personalbio Biotechnology Co., Ltd. (Shanghai, China) for untargeted metabolomics analysis [23,24]. The acquired raw metabolite peak intensity data were first subjected to log2 logarithmic transformation to stabilize variance, followed by unit variance (UV) scaling for normalization to eliminate interference from differences in metabolite abundance on subsequent statistical analyses. The preprocessed data were then processed with multivariate statistical analyses, including principal component analysis (PCA) and orthogonal partial least squares discriminant analysis (OPLS-DA). Specifically, PCA, an unsupervised dimensionality reduction approach based on the singular value decomposition (SVD) algorithm, was performed to intuitively visualize the differences in metabolic profiles between groups and the repeatability of intra-group samples. OPLS-DA was conducted using the orthogonal signal correction-partial least squares algorithm to maximize the discrimination of metabolic differences between groups by filtering out orthogonal noise signals unrelated to grouping. Meanwhile, a 200-time permutation test was applied to verify the stability of the OPLS-DA model and avoid model overfitting. Different metabolites were screened based on the Variable Importance in Projection (VIP) values calculated from the OPLS-DA model, in combination with *p*-values from Student’s *t*-test, with the screening threshold set as VIP > 1 and *p* < 0.05. The differential metabolites were visualized via a volcano plot and hierarchical clustering heatmap. The volcano plot was generated based on log2-transformed inter-group fold change (FC) and −log10-transformed *p-*values; for the hierarchical clustering heatmap, the abundance data of differential metabolites were first normalized by Z-score transformation, and then plotted via hierarchical clustering algorithm based on Euclidean distance. Finally, pathway enrichment analysis of the differential metabolites was performed against the KEGG database using the hypergeometric test algorithm to identify the metabolic pathways significantly perturbed by Cd exposure.

### 2.9. Transcriptomic Analysis

Mouse mammary gland tissue samples were collected and submitted to Shanghai Personalbio Biotechnology Co., Ltd. (Shanghai, China) for transcriptomic analysis [25]. After total RNA extraction, quality control, strand-specific library construction, and sequencing on an Illumina platform, raw sequencing data were subjected to quality filtering. Clean reads were aligned to the mouse reference genome for gene expression quantification, and a gene expression matrix was generated using Transcripts Per Million (TPM) as the normalized unit for subsequent analyses. Based on the preprocessed gene expression matrix, PCA was performed to verify inter-group differences and intra-group biological repeatability. Data were first subjected to log2 transformation and UV scaling, followed by unsupervised dimensionality reduction using the SVD algorithm. Genes with FDR < 0.05 adjusted by the Benjamini–Hochberg (BH) method and |log2 fold change (FC)| > 1 were defined as significantly differentially expressed genes. Differentially expressed genes were visualized by volcano plots and hierarchical clustering heatmaps. Volcano plots were constructed based on log2-transformed FC and −log10-transformed adjusted *p-*values; for hierarchical clustering heatmaps, TPM values of differentially expressed genes were normalized by Z-score transformation, followed by clustering using the Euclidean distance and complete linkage method. A hypergeometric test was performed for GO functional enrichment analysis and KEGG pathway enrichment analysis on the screened significantly differentially expressed genes. Gene Set Enrichment Analysis (GSEA) corresponding to GO and KEGG was conducted using the weighted enrichment algorithm based on the whole gene expression profile.

### 2.10. Statistical Analysis

All data are displayed as the mean ± standard error of the mean, or simply standard error (SEM). Plots were generated using the Student’s *t*-test (two groups) or the one-way analysis of variance (ANOVA; multiple groups) and Prism 11.0 (GraphPad, San Diego, CA, USA); *p*-values less than 0.05 were considered statistically significant. Each experiment has been conducted a minimum of three times, and representative images are provided.

## 3. Results

### 3.1. Network Toxicology Predicts Endocrine and Neuroendocrine Pathways Linking Cd Exposure to Lactation Impairment

To explore the potential molecular mechanisms by which Cd exposure may impair lactation, a network toxicology approach was applied. Cd-related genes were retrieved from the CTD, while genes associated with delayed onset of lactogenesis were collected from GeneCards. Intersection analysis identified 43 overlapping genes between the two datasets (Figure 1A), suggesting a potential molecular link between Cd exposure and lactation dysfunction. GO and KEGG pathway enrichment analyses revealed that these intersecting genes were predominantly enriched in endocrine- and lactation-related pathways, including prolactin signaling, growth hormone synthesis and secretion, janus kinase-signal transducer and activator of transcription (JAK-STAT) signaling, phosphatidylinositol 3-kinase-protein kinase B (PI3K-Akt) signaling, cyclic adenosine monophosphate (cAMP) signaling, and neuroactive ligand–receptor interaction (Figure 1B–D). Notably, pathways directly associated with mammary gland development and mammary epithelium development were also significantly enriched. PPI network analysis showed strong connectivity among these targets (Figure 1E), especially JAK-STAT signaling-related genes. Collectively, network toxicology analysis predicted that Cd exposure may impair lactation primarily through disruption of endocrine and neuroendocrine signaling pathways regulating mammary gland development and function.

### 3.2. Cd Exposure Induces Offspring Weight Loss

**Figure 2:**
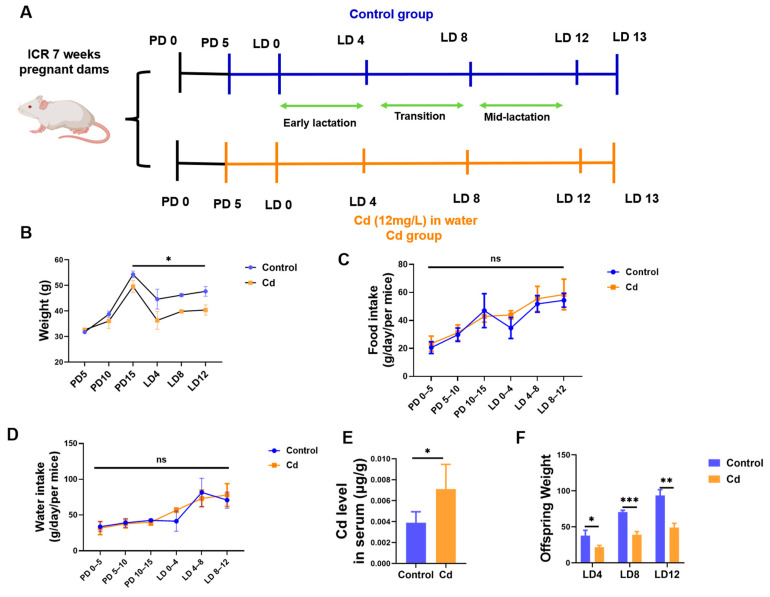
Effects of maternal Cd exposure on offspring growth. (**A**) Schematic of Animal experimental design. (**B**) Dams’ weight change. (**C**) Altered food intake and water consumption (**D**) in dams. (**E**) Altered Cd consumption in serum of dams. (**F**) Offspring weight change. “ns” indicates no significant difference, “*” indicates *p* < 0.05. “**” indicates *p* < 0.01. “***” indicates *p* < 0.001.

As illustrated in Figure 2A, female mice were exposed to Cd via drinking water during gestation and lactation. Compared with the Control group, Cd-exposed maternal mice exhibited significantly lower body weights during lactation periods (Figure 2B). However, it should be noted that there was no significant difference in both food and water intake between the two groups (Figure 2C,D). Our findings revealed that Cd administered through Cd-supplemented drinking water accumulated in the maternal serum. Cd concentrations were significantly elevated in the Cd group (*p* < 0.05, Figure 2E). Additionally, compared with the Control group, the offspring of Cd-treated dams showed significantly reduced body weights (*p* < 0.05, Figure 2F).

### 3.3. Cd Exposure Induces Mammary Morphology Abnormalities in Maternal Mammary Gland

**Figure 3:**
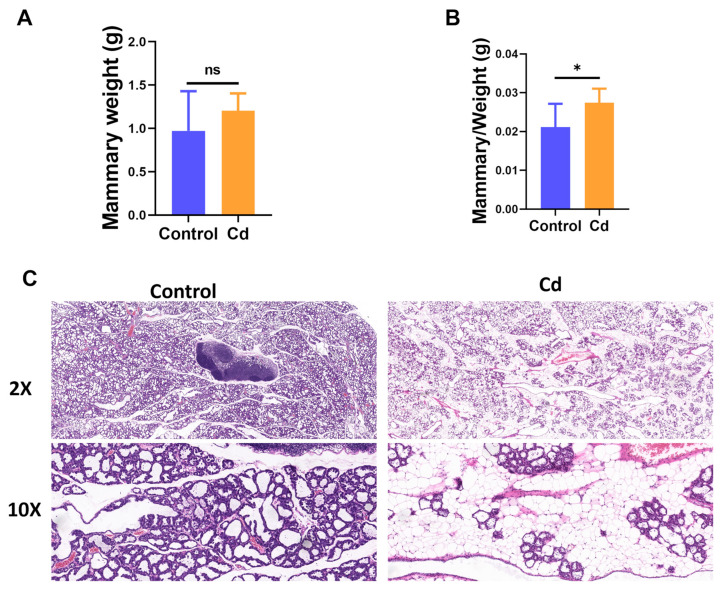
Maternal Cd exposure induces histopathological alteration in the mammary gland. (**A**) Mammary gland weight in dams. (**B**) Relative mammary gland weight index in dams. (**C**) Histopathological alterations of the mammary gland collected from LD13. Scale bar is 50 and 10 µm, respectively. “ns” indicates no significant difference, “*” indicates *p* < 0.05.

To determine whether the mammary gland is a direct target of Cd toxicity, mammary gland weight, mammary gland index, and histological morphology were evaluated. No significant differences were observed in maternal mammary gland weight (*p* > 0.05, Figure 3A), but there was a significant change in mammary gland index between the Cd and Control group (*p* < 0.05, Figure 3B). HE staining revealed distinct morphological alterations in the mammary gland following Cd exposure. Compared with the Control group, mammary tissue from the Cd group showed reduced alveolar density, disorganized acinar arrangement, and thinning of the epithelial lining with diminished secretion (Figure 3C). These histopathological abnormalities indicate that Cd exposure disrupts normal mammary gland architecture, which may underlie impaired lactational function during pregnancy.

### 3.4. Effect of Cd Exposure Caused Hormonal Homeostasis Disruption in Dams

**Figure 4:**
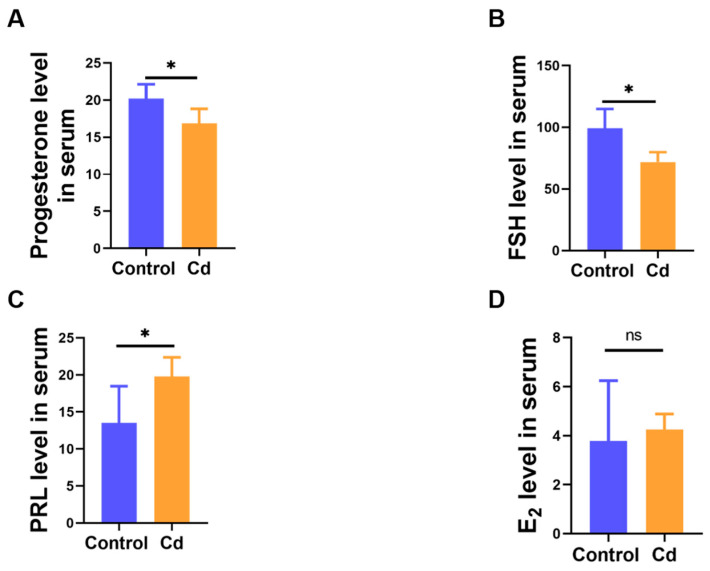
Reproductive hormone changes follow Cd treated in dams. The levels of progesterone (**A**), FSH (**B**), PRL (**C**), and E_2_ (**D**) in serum. “ns” indicates no significant difference, “*” indicates *p* < 0.05.

Given the essential role of endocrine regulation in mammary development and lactation, serum levels of key reproductive and lactation-related hormones were assessed. We confirmed that maternal Cd exposure resulted in a significant reduction in reproductive hormone levels. Compared with the Control group, serum concentrations of progesterone and FSH were significantly decreased, whereas PRL concentrations were markedly elevated in Cd-treated mice (*p* < 0.05, Figure 4A–C). However, there was no significant change between the Control and Cd group in E_2_ level (*p* > 0.05, Figure 4D). These findings indicate that chronic Cd exposure during gestation disrupts endocrine homeostasis by suppressing key reproductive and lactation-associated hormones.

### 3.5. Cd Impairs Lactation Performance and Milk Nutritional Quality

**Figure 5:**
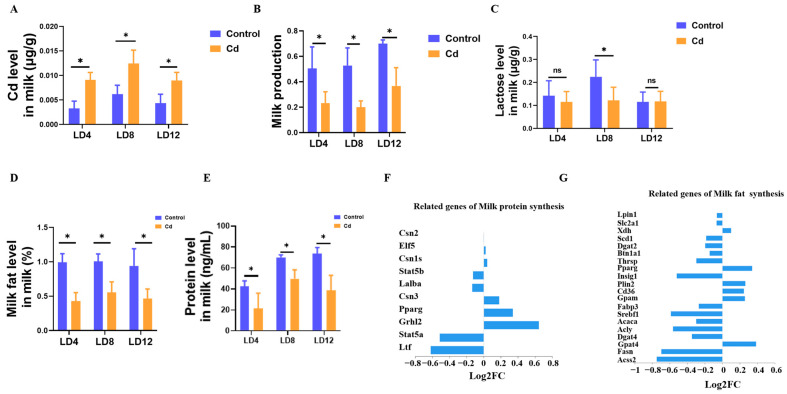
Lactating dams exhibited significant alterations in both milk production and compositional quality. (**A**) The level of Cd in the milk. (**B**) Milk production. The level of lactose (**C**), milk fat (**D**), and milk protein (**E**) in the milk. (**F**) Milk protein-related genes. (**G**) Milk fat-related genes. “ns” indicates no significant difference, “*” indicates *p* < 0.05.

To directly assess lactational performance, milk yield and milk composition were measured at early, transition, and mid-lactation stages. Cd concentrations in breast milk were significantly higher in Cd-exposed dams than in controls at all examined stages (*p* < 0.05, Figure 5A), confirming Cd transfer into milk. Compared with the Control group, dams exposed to Cd exhibited a significant reduction in milk yield throughout the entire lactation period (*p* < 0.05, Figure 5B). The decline was already evident during early lactation and became more pronounced at transition and mid-lactation stages. In addition to the reduction in milk production, the major nutritional components of milk were markedly affected. Similarly, milk protein and fat levels showed consistent declines at all examined lactation stages (*p* < 0.05, Figure 5D,E). In contrast, lactose levels displayed a stage-specific pattern. No significant difference was observed during early or mid-lactation, but a significant reduction was detected in late lactation (Figure 5C). To further explore the molecular basis of these observed changes in milk composition, we examined the level of key genes involved in milk protein and fat synthesis. Critically, milk protein synthesis genes (*Csn1s1*, *Csn2*, *Stat5a/b*) were suppressed, paralleled by broad inhibition of milk fat synthesis regulators, including *Srebf1*, *Fasn*, and *Pparg*, following Cd treatment (Figure 5F,G).

### 3.6. Cd Induces Global Metabolic Reprogramming in the Mammary Gland

**Figure 6:**
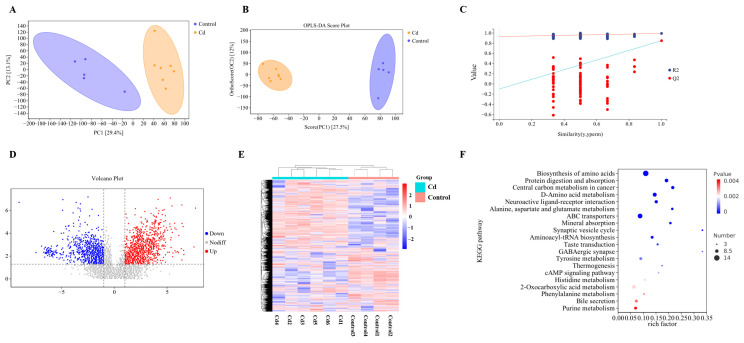
Metabolite identification in mammary tissue. (**A**) PCA score plot (unsupervised). (**B**) OPLS-DA plot. (**C**) Validation plot (permutation test) for OPLS-DA model stability. (**D**) Volcano plot of differential metabolites. (**E**) Clustering heatmap of total metabolites. (**F**) KEGG factor plot.

To gain a clearer understanding of the metabolic variation patterns in mammary gland tissues under Cd exposure, we performed a non-targeted metabolomics analysis of breast tissue using liquid chromatography-tandem mass spectrometry (LC-MS/MS). The PCA plot revealed a clear separation between the Cd group and the Control group, indicating distinct metabolic profiles induced by Cd treatment (Figure 6A). Furthermore, samples clustered closely within their respective groups, demonstrating good experimental reproducibility. To further maximize the inter-group differences and identify key differential metabolites, a supervised OPLS-DA was employed. The OPLS-DA plot demonstrated a more distinct separation between the Cd and Control groups compared to PCA (Figure 6B). Model robustness was confirmed by a 200-times permutation test, yielding high R^2^Y (0.991) and Q^2^ (0.848) values, with no evidence of overfitting (*p* < 0.05, Figure 6C). Differential metabolite analysis identified a total of 130 significantly altered metabolites between the two groups, including 93 upregulated and 37 downregulated metabolites in the Cd group (Figure 6D and Table S1). As shown in the heatmap, all selected metabolites exhibited distinct group-specific clustering. Samples from the Cd group and the Control group were clearly separated, indicating that Cd exposure induced a global reprogramming of the metabolomic profile in mammary tissue (Figure 6E). To explore the biological relevance of these metabolic alterations, KEGG pathway enrichment analysis was performed. A total of 53 metabolic pathways were significantly enriched (Table S2), among which the top 20 pathways are visualized (Figure 6F). These pathways were mainly associated with amino acid metabolism, neuroactive ligand–receptor interaction, mineral absorption, tyrosine metabolism, energy metabolism, and hormone-related signaling, indicating that Cd exposure induces broad metabolic disturbances in the mammary gland.

### 3.7. Cd Disrupts Steroid Hormone, Neuroendocrine, and Amino Acid Metabolism Related to Lactation

**Figure 7:**
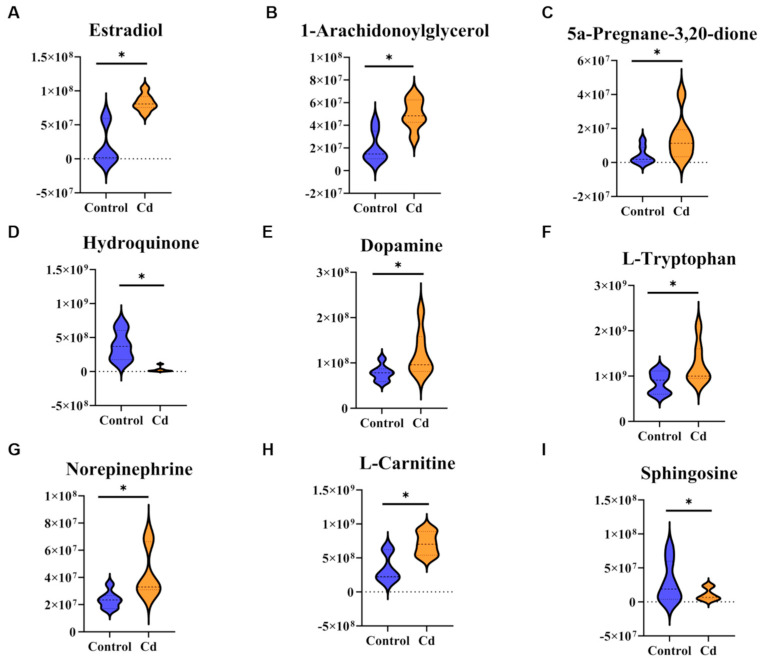
Differential metabolites related to lactation function and hormone levels. (**A**) Estradiol. (**B**) 1-Arachidonoylglycerol. (**C**) 5a-Pregnane-3,20-dione. (**D**) Hydroquinone. (**E**) Dopamine. (**F**) L-Tryptophan. (**G**) Norepinephrine. (**H**) L-Carnitine. (**I**) Sphinganine. “*” indicates *p* < 0.05.

Integrated metabolomic analyses revealed that the mammary gland following Cd exposure was altered, particularly affecting pathways involved in hormone synthesis, neuroendocrine signaling, amino acid metabolism, and energy production. In steroid hormone metabolism, several key lactation-related metabolites were significantly altered. Compared with the Control group, levels of estradiol, 1-arachidonoylglycerol, and 5α-pregnane-3,20-dione were markedly decreased in the Cd group, whereas hydroquinone was significantly increased (*p* < 0.05, Figure 7A–D). These alterations indicate disruption of steroid hormone biosynthesis and redox balance, consistent with endocrine dysregulation observed at the systemic level. Neuroendocrine-related metabolic pathways were also prominently affected. Cd also depressed the level of dopamine, L-tryptophan, norepinephrine, and L-carnitine were downregulated, while sphinganine was elevated (*p* < 0.05, Figure 7E–I). Together, these findings suggest impaired neurotransmitter synthesis and signaling, indicating disruption of neuroendocrine regulation within the hypothalamic–pituitary–mammary axis.

**Figure 8:**
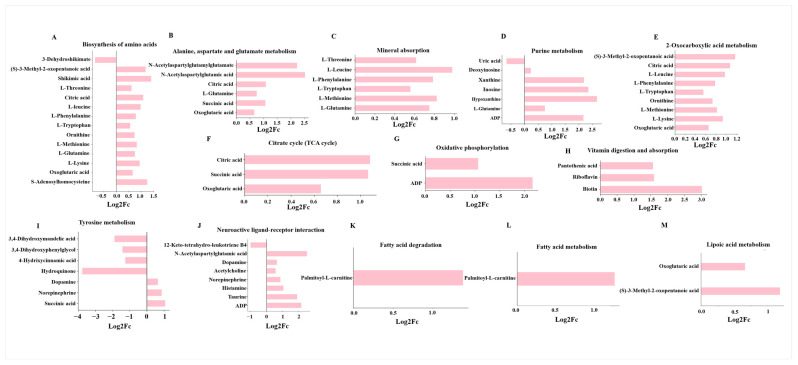
Differential metabolites are involved in biological processes associated with sex hormone levels and lactation function. (**A**) Biosynthesis of amino acids. (**B**) Alanine, aspartate, and glutamate metabolism. (**C**) Mineral absorption. (**D**) Purine metabolism. (**E**) 2-Oxocarboxylic acid metabolism. (**F**) TCA cycle. (**G**) Oxidative phosphorylation. (**H**) Vitamin digestion and absorption. (**I**) Tyrosine metabolism. (**J**) Neuroactive ligand–receptor interaction. (**K**) Fatty acid degradation (**L**) Fatty acid metabolism. (**M**) Lipoic acid metabolism.

In pathways related to amino acid biosynthesis and alanine, aspartate, and glutamate metabolism, levels of L-threonine, L-leucine, and L-glutamine were significantly elevated, suggesting either compensatory accumulation or impaired utilization for milk protein synthesis (Figure 8A,B). Consistently, the mineral absorption and purine metabolism pathways were significantly affected, with elevated L-Leucine, ADP, and hypoxanthine, pointing to nucleotide turnover imbalance. Disruptions in 2-oxocarboxylic acid metabolism with accumulation of 3-Methyl-2-oxopentanoic acid indicated metabolic stalling between amino acid catabolism and the tricarboxylic acid (TCA) cycle (Figure 8C–E). Key intermediates of the TCA cycle, including succinic acid and oxoglutaric acid, were elevated alongside increased ADP levels, suggesting partial blockage of the TCA cycle and inefficient oxidative phosphorylation (Figure 8F,G). Cd exposure also altered vitamin digestion and absorption, as evidenced by elevated pantothenic acid, riboflavin, and biotin, implying impaired utilization of cofactors necessary for enzymatic reactions within oxidative metabolism (Figure 8H). The tyrosine metabolism pathway was markedly altered, with hydroquinone accumulation, reflecting catecholamine synthesis inhibition and oxidative stress (Figure 8I). In the neuroactive ligand–receptor interaction pathway, acetylcholine, taurine, and histamine were elevated (Figure 8J). Elevated levels of palmitoyl-L-carnitine and oxoglutaric acid indicated incomplete mitochondrial β-oxidation and lipid metabolic inefficiency (Figure 8K–M). In addition, suppression of the peroxisome proliferator-activated receptor (PPAR) signaling pathway was observed, supported by downregulation of 12-keto-tetrahydro-leukotriene B4 (Figure S1D), a lipid mediator involved in fatty acid oxidation and milk fat synthesis.

### 3.8. Cd Induces a Predominantly Inflammatory Transcriptional Profile in the Mammary Gland

**Figure 9:**
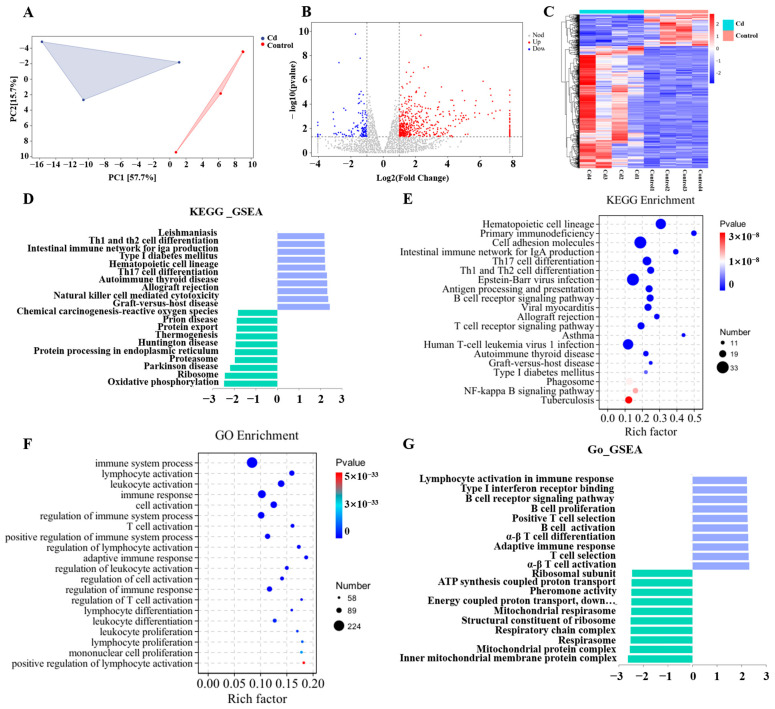
Alterations in the transcriptomic characteristics of mammary tissue upon Cd exposure. (**A**) PCA plot of transcriptomic profiles. (**B**) Volcano plot of differentially expressed genes in Cd-exposed versus Control mice. (**C**) Heatmap of differentially expressed genes. (**D**) Key pathways derived from GSEA following KEGG analysis. (**E**) Significantly enriched KEGG pathways identified by enrichment analysis. (**F**) Significantly enriched GO terms from Gene Ontology analysis. (**G**) Essential pathways identified by GSEA after GO term analysis.

To investigate transcriptomic alterations in the mammary gland induced by Cd exposure, RNA sequencing was performed on mammary gland tissues from the Control and Cd groups. RNA-seq analysis displayed distinct clustering of mammary transcriptomes between Cd and Control groups (Figure 9A). Differential expression analysis identified 441 significantly upregulated and 114 downregulated genes, with hierarchical clustering revealing polarized expression patterns between Cd-enriched and control-enriched gene clusters (Figure 9B,C). GSEA pathway analysis demonstrated pronounced immune activation, showing upregulated hematopoietic cell lineage, T cell receptor signaling, and Nuclear Factor-kappa B (NF-κB) signaling, alongside downregulated oxidative phosphorylation and ribosome biogenesis (Figure 9D). KEGG analysis further confirmed immune dysregulation, highlighting allograft rejection, B cell receptor signaling, and NF-κB pathway activation (Figure 9E and Table S3). GO analysis corroborated these findings, revealing enriched lymphocyte activation and immune response regulation (Figure 9F), while suppressing mitochondrial ATP synthesis and protein modification (Figure 9G).

### 3.9. Cd Disrupts Hormone Signaling in the Mammary Gland

**Figure 10:**
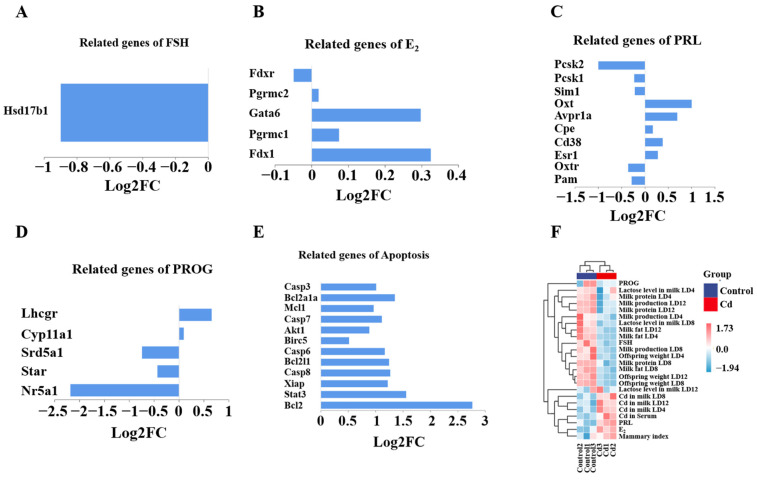
DEGs induced by Cd exposure were involved in and led to impaired maternal hormone levels in the dams. (**A**) FSH-related genes. (**B**) E_2_-related genes. (**C**) PRL-related genes. (**D**) Progesterone-related genes. (**E**) Apoptosis-related genes. (**F**) Correlation heatmap.

To further elucidate the impact of Cd exposure on mammary gland function, we specifically examined hormone- and lactation-related genes among the differentially expressed genes (DEGs). Transcriptomic analysis revealed Cd-induced dysregulation of key hormone-responsive and lactation-associated genes. FSH-related *Hsd17b1* was significantly downregulated, while E_2_-regulated genes (*Fdx1*, *Pgrmc1*, *Gata6*) exhibited mixed changes, with *Fdx1* showing the strongest upregulation in Cd-treated mice (Figure 10A,B). Compared with the Control group, PRL signaling genes displayed polarized responses, as *Esr1* and *Oxtr* were suppressed, whereas *Pcsk1* and *Cd38* were higher (Figure 10C). Progesterone-related genes were also universally downregulated in the Cd group (Figure 10D). Conversely, compared with the Control group, apoptosis genes (*Casp3*, *Casp8*, *Bcl2*) were markedly activated in the Cd group (Figure 10E). To integrate molecular alterations with physiological and developmental outcomes, correlation analysis was performed across Cd exposure indices, hormonal profiles, lactation parameters, and offspring growth metrics. Hierarchical clustering revealed a clear segregation between Cd levels in serum- and lactation-associated parameters, including milk yield, milk protein, milk fat, lactose content, and offspring body weight (Figure 10F). Notably, progesterone and FSH clustered positively with lactation indices, whereas PRL and estradiol exhibited an opposing correlation pattern. These results demonstrate a coordinated disruption of hormone homeostasis and lactation-related gene expression associated with Cd exposure, providing an integrated molecular framework linking Cd burden to impaired mammary function and compromised offspring growth.

## 4. Discussion

**Figure 11:**
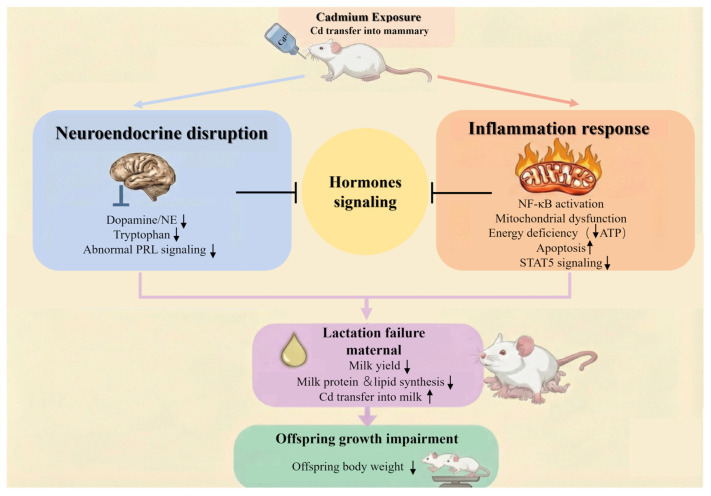
Cd impaired lactation capacity by hormone signaling dysfunction in the mammary gland of mice. Cd impaired lactation capacity by hormone signaling dysfunction in the mammary gland of mice. ↑ means upregulation, ↓ means downregulation.

Here, we established a mouse model of Cd exposure during gestation and lactation to investigate its impact on mammary gland function and lactational performance. Using a drinking water exposure paradigm that closely reflects environmental exposure scenarios, we demonstrated that maternal Cd exposure led to reduced maternal and offspring body weight, impaired mammary gland development, disrupted lactation-related hormonal homeostasis, and markedly compromised milk yield and nutritional composition (Figure 11). These alterations were accompanied by coordinated metabolic and transcriptional disturbances in mammary tissue. Our findings provide novel insights into the potential mechanisms by which Cd acts as an environmental endocrine disruptor to compromise maternal and infant health.

Previous studies have reported that Cd exposure can adversely affect mammary physiology. However, most experimental investigations have relied on acute or relatively high-dose exposure paradigms, frequently employing intraperitoneal administration, and have largely overlooked the critical gestational and lactational windows during which the mammary gland undergoes dynamic remodeling [26,27,28]. While such approaches are valuable for examining acute toxic effects, they may not adequately reflect real-world exposure scenarios, in which Cd intake typically occurs through chronic oral ingestion of contaminated water or food. The present study established Cd exposure through drinking water across both gestation and lactation, thereby mimicking chronic oral exposure during biologically vulnerable stages of mammary development and lactation initiation. The 12 mg/L of Cd concentration used in this study falls within the lower range of doses reported in experimental drinking water models [28,29]. Previous studies have employed a broad range of Cd concentrations depending on the biological endpoints investigated, with higher exposure levels frequently used in studies focusing on Cd-induced neurotoxicity or systemic toxicity. Notably, the Cd concentration used in this study is comparable to levels reported in groundwater from mining-contaminated regions in China, highlighting the environmental relevance of our exposure design [28,29]. Environmental monitoring studies have reported elevated Cd concentrations in groundwater and drinking water sources in certain mining-impacted regions of China, where long-term contamination has resulted in persistent Cd exposure for local populations. These findings highlight the potential for chronic Cd intake through drinking water and underscore the importance of investigating its biological consequences under environmentally relevant exposure scenarios. Using this drinking water Cd exposure model, we observed significant Cd accumulation in maternal serum and mammary tissue, accompanied by suppressed maternal weight gain during lactation and pronounced growth retardation in offspring, despite unaltered food and water intake. According to the latest toxicological study by Kim et al., mice exposed to drinking water containing Cd showed no significant differences in body weight gain, total food intake, or average daily water consumption compared with the control group [30]. No obvious clinical toxic symptoms or acute lethality were observed. This dose ensures the basic physiological survival of maternal mice during gestation and lactation, thereby providing an ideal experimental window for investigating subtle endocrine-disrupting effects induced by Cd exposure. These findings indicate that chronic oral Cd exposure (12 mg/L) exerts direct toxic effects on both dams and offspring. This methodological distinction is critical, as it allows our findings on mammary gland dysfunction and impaired lactation to be interpreted in a context that more closely resembles real-world exposure scenarios. Therefore, our model provides more translationally relevant insights into how environmental Cd contamination may compromise maternal lactation performance and offspring growth.

In our study, the mammary weight index was increased in Cd-treated dams. Histological analysis further revealed that Cd exposure led to reduced alveolar density, disorganized structure, and thinning of the epithelial lining in the mammary gland. The mammary gland is a highly dynamic organ that undergoes a second wave of development during pregnancy and lactation, mediated by various reproductive hormones [13,31,32]. Increasing evidence suggests that E_2_ plays a central role in regulating ductal outgrowth and branching morphogenesis. Progesterone and PRL collaborate to drive alveolar proliferation and maturation. FSH indirectly contributes by stimulating ovarian steroid hormones, ensuring an adequate supply of E_2_ and progesterone [13,33]. Disruption of this coordinated hormonal network during the gestational–lactational window may therefore have important consequences for mammary gland development and lactational competence. Cd is a known environmental endocrine disruptor that triggered a complex hormonal imbalance in previous studies [34,35]. At the level of neuroendocrine regulation, research by Bhardwaj et al. demonstrated that Cd exposure at this concentration significantly downregulates the gene expression of hypothalamic Kiss1 and its receptor Kiss1r, thereby interfering with the functional maturation of gonadotropin-releasing hormone (GnRH) neurons [36]. Recent evidence from Santiago-Andres et al. indicated that Cd exhibits significant bioaccumulation in the pituitary gland and induces gonadotroph hyperplasia [37]. By disrupting the delicate intracellular Ca^2+^ signaling pattern, Cd disturbs the secretory rhythms of LH and FSH. With regard to the mammary gland as a target organ, a study by Li et al. revealed that Cd exposure suppresses the self-renewal capacity of mammary epithelial stem cells via activation of the SHH signaling pathway, and significantly downregulates the expression of key milk synthesis genes such as β-casein [38]. Ultimately, these effects impair the lactation function of maternal mice at both histological remodeling and molecular synthesis levels. In our study, the levels of progesterone and FSH were significantly decreased in the Cd group, which may contribute to impaired alveologenesis and lobuloalveolar differentiation. Consistently, Hsd17b1, a key enzyme involved in steroid hormone biosynthesis, showed reduced expression in Cd-treated mammary tissue, which may be associated with the observed decline in circulating FSH and progesterone. These findings suggest that Cd exposure may interfere with endocrine signaling involved in mammary gland development. Moreover, Cd exposure was associated with abnormally elevated levels of PRL, while the expression of receptor-related genes, including Oxtr, was markedly reduced. Li et al. reported that dysregulated PRL signaling may influence progesterone homeostasis and affect STAT5 activation, thereby potentially impairing lactogenic differentiation [39,40]. Notably, transcriptomic data revealed the downregulation of *Esr1*, which may represent a compensatory response of cells to the expression of elevated E_2_, ultimately desensitizing mammary tissue to lactogenic signals and inhibiting milk ejection. The expression of hormones and genes depresses the structural and functional competence of the mammary gland for lactation. It should be noted that the present study did not directly examine the hypothalamus or pituitary glands. Therefore, although significant alterations in circulating hormone levels were observed, the involvement of the central hypothalamic–pituitary regulatory axis cannot be definitively established. Previous studies have reported that Cd can cross the blood–brain barrier and interfere with neuroendocrine regulation, while other studies indicate that Cd exposure may impair ovarian steroidogenesis, leading to altered systemic hormone levels. These findings suggest that the endocrine alterations observed in this study may originate from multiple regulatory sites, including the ovary and neuroendocrine centers. Future studies directly examining these tissues will be necessary to clarify the precise mechanisms underlying Cd-induced lactational dysfunction. Consistent with these experimental observations, network-based analysis further highlighted prolactin signaling, STAT 5 signaling, and hormone-regulated mammary development pathways as key pathways associated with Cd exposure, supporting a mechanistic link between hormonal disruption and lactation failure.

Our study provides direct evidence of the detrimental effects of Cd exposure on lactation performance. On one hand, Cd exposure induced a robust inflammatory transcriptional program in the mammary gland, characterized by activation of immune-related pathways and concomitant suppression of oxidative phosphorylation and ribosome biogenesis. Integrated transcriptomic pathway enrichment analysis revealed that these differentially expressed genes were predominantly clustered in immune and metabolic regulatory pathways, indicating a coordinated inflammatory response. Sustained inflammation imposes a substantial energetic burden, and consistent with this, metabolomic profiling revealed impaired mitochondrial energy metabolism, including TCA cycle disruption, accumulation of ADP, and reduced ATP-generating capacity [41]. Integration of metabolomic and transcriptomic datasets further suggested that inflammatory signaling and mitochondrial metabolic dysfunction were closely interconnected, highlighting a systems-level metabolic constraint on lactation. Such energy deprivation is incompatible with the high biosynthetic demands of lactation. Prolonged metabolic stress and inflammatory activation further culminated in enhanced apoptotic signaling within mammary epithelial cells, as evidenced by the upregulation of *Casp3*, *Casp8*, and *Bcl2* [42]. This increase in programmed cell death likely reduces the functional mammary epithelial cell pool, thereby directly into impaired milk synthesis. Notably, Cd exposure also inhibited key milk protein synthesis genes and milk fat synthesis genes, providing genetic evidence for the observed decline in milk yield and nutritional content. Network-based integration of differentially expressed genes further indicated that these milk synthesis-related genes were functionally connected with inflammatory and metabolic pathways, suggesting that Cd exposure may disrupt lactational capacity through coordinated pathway-level alterations rather than isolated molecular events. Consistent with gene suppression, reduced alveolar density and widened interstitial spaces were observed in the Cd mammary gland. In addition to reduced milk quantity and quality, elevated Cd concentrations detected in milk suggest a potential route of direct metal transfer from dams to offspring. The mammary gland, therefore, not only functions as a target organ of Cd toxicity but also serves as a conduit for maternal and offspring Cd transmission. Prenatal Cd exposure has been shown to induce intrauterine growth restriction by disrupting placental development and nutrient transport [43,44]. Consistent with these reports, the reduced offspring body weight observed in our study likely reflects the combined impact of compromised fetal growth during gestation and insufficient nutritional support during the postnatal period. On the other hand, Cd accumulation within mammary tissue and milk likely perturbs neuroendocrine regulation of lactation [45,46,47]. Integrated metabolomic and transcriptomic analyses suggested that Cd induced significant reductions in dopamine, norepinephrine, and L-tryptophan, which suggests disruption of neuroendocrine pathways involved in prolactin regulation and milk ejection. Integrated pathway enrichment analysis further highlighted neuroactive ligand receptor interaction and prolactin signaling pathways as key nodes potentially affected by Cd exposure. This neuroendocrine interference may further compromise lactational performance by impairing signal transmission along the hypothalamic–pituitary–mammary axis [48]. Notably, these two pathological axes converge on the suppression of STAT5-dependent signaling, a central transcriptional driver of mammary differentiation and milk protein synthesis [49,50]. These analyses consistently highlighted alterations in pathways related to prolactin signaling, energy metabolism, and mammary gland development, supporting a coordinated disruption of regulatory networks involved in lactational function. Although targeted validation experiments such as qPCR or Western blotting were not performed in the present study, the key pathways identified here were supported by multiple independent lines of evidence, including transcriptomic profiling, metabolomic alterations, and histological changes in mammary tissue. Future studies focusing on targeted molecular validation will help further clarify the specific regulatory mechanisms underlying Cd-induced lactational dysfunction.

## 5. Conclusions

This study demonstrated that Cd exposure through drinking water during pregnancy significantly disrupts maternal endocrine balance, impairs mammary gland development, and reduces both milk yield and its key nutritional components. The observed suppression of pregnancy-related hormones, combined with histopathological damage to mammary tissue, suggests that Cd acts as an environmental endocrine disruptor that compromises lactation capacity. Multi-omics analyses further revealed coordinated alterations in inflammatory, metabolic, and hormone-related signaling pathways that may contribute to disrupted mammary gland function. A major strength of this study is the integration of physiological measurements, histological evaluation, and combined transcriptomic and metabolomic analyses, providing a systems-level perspective on how Cd exposure may influence lactational function. Our findings provide novel experimental evidence for the adverse effects of Cd on lactation and underscore the need for strengthened environmental health risk assessment, preventive strategies, and targeted interventions to safeguard mammary development well-being.

## Data Availability

Data will be made available on request.

## Supplementary Materials

The following supporting information can be downloaded at https://www.mdpi.com/article/10.3390/biology15100754/s1: Figure S1: Differential metabolites are involved in signaling pathways associated with sex hormone levels and lactation function. (A). mTOR signaling pathway. (B). cAMP signaling pathway. (C). FoxO signaling pathway (D). PPAR signaling pathway. (E). Glucagon signaling pathway. (F). Prolactin signaling pathway; Table S1: Differential metabolites between the Cd and the Control group in mammary; Table S2: Differential metabolites in various pathways were enriched; Table S3: Identified differential genes from transcriptomic analyses of mammary tissue.
