# Supplementary material for: Maternal Cadmium Exposure Impairs Lactational Performance and Milk Quality in Mice
Source: Biology (Basel). 2026 May 9;15(10):754. doi: 10.3390/biology15100754 (PMC13203474; doi:10.3390/biology15100754)
Supplement: Supplementary file 1 [file biology-15-00754-s001.zip › Table S1.pdf]

Table S1. Differential metabolites between the Cd and the Control group in mammary tissue

ID	log2FoldChange	Pvalue	FDR	vip	Regulation
M102T589	4.560362366	0.003873	0.064515	1.682638	Up
M102T62	4.147125161	0.017514	0.123946	1.546584	Up
M102T685	3.284252691	3.01E-06	0.001856	2.159352	Up
M107T409	3.018453378	0.003842	0.06424	1.688224	Up
M109T305	2.717296922	0.040142	0.181229	1.316748	Up
M110T294	2.661796407	0.000318	0.020864	1.953562	Down
M110T641	2.657463716	6.29E-05	0.00874	2.066793	Down
M111T508	2.64425921	0.000148	0.014128	1.988868	Down
M112T42	2.523832055	0.041299	0.1837	1.262364	Up
M113T33	2.417230151	0.001631	0.044691	1.924006	Up
M114T196	2.347190475	0.035424	0.170993	1.33245	Down
M114T50	2.344656999	0.007463	0.082335	1.571053	Up
M115T43	2.230033587	0.018509	0.12738	1.478176	Up
M115T99	2.180976981	0.010005	0.094993	1.535114	Up
M117T41_2	2.172719522	0.025384	0.389601	1.68743	Up
M120T49	2.164903383	0.037766	0.17623	1.357672	Up
M126T43	2.160874959	0.015924	0.118285	1.584992	Up
M126T50	1.995363991	0.002447	0.052137	1.729033	Up
M127T229	1.923051458	0.000234	0.018071	1.897744	Up
M129T221	1.909833192	0.001653	0.044731	1.739832	Up
M129T65	1.846341401	0.039052	0.404094	1.511236	Up
M130T188	1.838351996	0.011733	0.102279	1.546081	Up
M130T49	1.832901524	0.011583	0.348603	1.774559	Up
M130T56_1	1.800861971	0.045298	0.191948	1.285278	Up
M131T34	1.654726886	0.036922	0.174306	1.340666	Down
M132T240	1.613963705	0.007523	0.082648	1.608894	Up
M132T36	1.597506081	2.64E-05	0.006207	2.064993	Down
M133T43	1.595823773	0.020446	0.133451	1.467743	Up
M133T62_1	1.565725273	0.003227	0.060321	1.786849	Up

M135T463	1.498418524	0.00112	0.037534	1.843261	Down
M135T477	1.481724272	0.008444	0.331382	1.799158	Down
M135T60	1.454310844	0.012716	0.353286	1.853288	Up
M135T76	1.454055486	0.002426	0.263329	2.229117	Up
M136T64	1.401222123	0.032728	0.164139	1.33744	Up
M139T390	1.378153301	0.007505	0.082621	1.622869	Up
M139T55	1.316654585	0.014115	0.11198	1.46842	Up
M145T47	1.311940539	0.024478	0.389601	1.714931	Up
M145T638	1.244317109	0.01691	0.121816	1.498068	Down
M146T188	1.228234603	0.012306	0.104468	1.510537	Up
M146T51	1.219199313	0.026109	0.147628	1.379231	Up
M147T43	1.196396575	0.003533	0.062093	1.674149	Up
M147T672	1.180097499	0.046465	0.19448	1.254908	Down
M150T62	1.170054872	0.003651	0.062879	1.777048	Up
M151T57	1.153754708	0.029395	0.389812	1.671628	Up
M157T35_1	1.142281428	0.03032	0.158951	1.356147	Down
M159T235	1.129511641	0.043721	0.404094	1.448359	Up
M159T62	1.081399848	0.049429	0.406906	1.334138	Up
M161T44	1.064147621	0.029518	0.156957	1.441999	Up
M162T411	1.060050388	0.013256	0.108476	1.54749	Down
M162T50	1.050206331	0.005929	0.07439	1.687284	Up
M162T56	1.047338936	0.036907	0.174276	1.344452	Up
M163T36	1.04204949	0.040362	0.181631	1.287784	Up
M163T409	1.027157569	0.006003	0.074679	1.632743	Up
M165T64	1.018134734	0.032364	0.16353	1.345222	Up
M165T88	0.979813194	0.008285	0.331367	2.098888	Up
M166T80	0.978763122	0.013734	0.110331	1.476904	Up
M167T47	0.957336901	0.040959	0.404094	1.467211	Down
M167T483	0.948616006	0.000363	0.022071	1.942698	Up
M168T103	0.943691533	0.009523	0.092866	1.55566	Up
M169T44	0.942865341	0.008608	0.088404	1.566908	Up

M169T95	0.938364252	0.00452	0.068634	1.665886	Up
M170T35	0.926658189	0.00716	0.081353	1.582018	Down
M170T484	0.886458859	0.004859	0.070071	1.743149	Up
M172T36	0.859967913	0.032217	0.163093	1.383417	Down
M173T165	0.823423517	0.049507	0.407174	1.446716	Up
M173T40	0.822830782	0.048087	0.406084	1.416762	Up
M176T329	0.800280536	0.02767	0.152472	1.428601	Up
M177T33	0.784975576	0.016284	0.119867	1.529279	Down
M178T195	0.779440259	0.012912	0.107299	1.474007	Up
M179T455	0.747489048	0.000294	0.020229	1.944614	Up
M182T64	0.741553619	0.003196	0.287447	1.863498	Up
M183T229	0.734815788	0.035281	0.170515	1.328792	Down
M183T353	0.734498081	0.0106	0.097528	1.553832	Down
M183T37_1	0.692695542	0.015807	0.117974	1.453327	Down
M183T64	0.657555783	0.030072	0.158279	1.36141	Up
M184T514	0.640913212	0.008414	0.087326	1.602244	Up
M185T36_2	0.640617404	0.000205	0.016772	2.05169	Down
M187T81	0.638700634	0.015563	0.117063	1.48172	Down
M188T271	0.636822067	0.025849	0.146994	1.406069	Up
M188T43	0.62659698	0.029131	0.156162	1.3251	Up
M189T45	0.615273891	0.020686	0.134015	1.45137	Up
M190T213	0.588699659	0.037504	0.175883	1.285185	Up
M191T38	0.57624551	0.03713	0.402967	1.570209	Up
M195T103	0.56685387	0.047392	0.196204	1.242731	Down
M195T272	0.561293142	0.029769	0.157718	1.347193	Up
M200T87	0.557901097	0.015275	0.116048	1.538085	Up
M204T284	0.492009976	0.02935	0.156686	1.446226	Up
M205T188	0.44250686	0.036451	0.173129	1.305695	Up
M215T321	0.438154508	0.007292	0.32892	1.976256	Down
M218T80	0.43412803	0.016898	0.121773	1.466946	Up
M219T55	0.397418026	0.047114	0.195449	1.336683	Up

M220T110	0.242500736	0.008998	0.090663	1.575479	Up
M224T33	0.229135276	0.01221	0.104091	1.51254	Down
M230T52	-0.547144687	0.035873	0.171805	1.36634	Up
M243T314	-0.582585128	0.001802	0.046196	1.768155	Up
M245T257	-0.648490877	0.003101	0.059137	1.707497	Up
M252T344	-0.662786551	0.028257	0.389812	1.811627	Up
M267T64_1	-0.726982058	0.015177	0.367449	1.812224	Up
M270T476	-0.768372528	0.026795	0.389601	1.79653	Down
M273T411	-0.779210469	0.012038	0.103436	1.644126	Up
M291T426	-0.838270339	0.047791	0.406084	1.554091	Down
M294T497	-0.850530519	0.026249	0.389601	1.717368	Down
M295T228	-1.009619651	0.031588	0.161701	1.414061	Up
M301T645	-1.022638989	0.04778	0.406084	1.591992	Down
M305T64	-1.104358212	0.003166	0.059913	1.750872	Up
M307T597	-1.112454959	0.027009	0.150597	1.376215	Down
M309T618	-1.16262246	0.040735	0.182428	1.261188	Down
M311T652	-1.196635411	0.007171	0.081358	1.548142	Down
M316T388_1	-1.198351382	0.023855	0.141867	1.500082	Up
M317T461	-1.260683627	0.006106	0.32892	2.045381	Down
M317T511	-1.289906893	0.009442	0.338471	1.768894	Down
M317T582	-1.421329722	0.026525	0.149038	1.351241	Up
M319T467	-1.525973324	0.017228	0.369869	1.848285	Down
M320T249	-1.594955351	0.031824	0.162136	1.344541	Up
M345T507	-1.673460167	0.030138	0.389812	1.697836	Down
M357T451	-1.830474781	0.032994	0.164621	1.335616	Up
M361T485	-1.884466338	0.003057	0.058839	1.779024	Up
M376T505	-1.993964406	0.049834	0.201185	1.249849	Down
M377T251_2	-2.15465387	0.010042	0.09516	1.519899	Up
M384T572	-2.18767883	0.002549	0.053066	1.748383	Up
M385T53	-2.252255862	0.041799	0.18457	1.342155	Up
M386T370	-2.31413386	0.013173	0.108138	1.51425	Down

M391T561	-2.356627328	0.004743	0.069246	1.65125	Up
M399T251	-2.499710716	0.024259	0.143058	1.342015	Up
M400T502_2	-2.758912993	0.015267	0.116048	1.418426	Up
M428T62	-2.866879891	0.011757	0.102291	1.617169	Up
M429T659_1	-3.467056792	0.001432	0.041934	1.761654	Up
M432T36	-3.762176545	0.007303	0.32892	2.01874	Up
M567T498_2	-3.838711196	0.005504	0.073034	1.644233	Up
M610T678	-4.519006298	0.011491	0.101439	1.515153	Up
