# Supplementary material for: Maternal Cadmium Exposure Impairs Lactational Performance and Milk Quality in Mice
Source: Biology (Basel). 2026 May 9;15(10):754. doi: 10.3390/biology15100754 (PMC13203474; doi:10.3390/biology15100754)
Supplement: Supplementary file 1 [file biology-15-00754-s001.zip › Table S2.pdf]

Table S2. Differential metabolites in various pathway were enriched

Pathway ID	Pathway	Up	Down	DEM	Total	P	FDR
mmu01230	Biosynthesis of amino acids	13	1	14	128	2.29E-07	2.02E-05
mmu04974	Protein digestion and absorption	9	0	9	47	3.20E-07	2.02E-05
mmu05230	Central carbon metabolism in cancer	8	0	8	37	5.62E-07	2.36E-05
mmu00470	D-Amino acid metabolism	8	2	10	69	1.04E-06	3.28E-05
mmu04080	Neuroactive ligand-receptor interaction	7	1	8	53	9.85E-06	0.000248
mmu00250	Alanine, aspartate and glutamate metabolism	6	0	6	28	1.69E-05	0.000331
mmu02010	ABC transporters	11	1	12	138	2.03E-05	0.000331
mmu04978	Mineral absorption	6	0	6	29	2.10E-05	0.000331
mmu04721	Synaptic vesicle cycle	4	0	4	12	7.36E-05	0.000989
mmu00970	Aminoacyl-tRNA biosynthesis	7	0	7	52	7.85E-05	0.000989
mmu04742	Taste transduction	5	0	5	32	0.000431	0.00494
mmu04727	GABAergic synapse	3	0	3	9	0.00065	0.006826
mmu00350	Tyrosine metabolism	3	4	7	78	0.001004	0.009733
mmu04714	Thermogenesis	4	0	4	23	0.001107	0.00996
mmu04024	cAMP signaling pathway	4	0	4	25	0.001532	0.012869
mmu00340	Histidine metabolism	5	0	5	47	0.002577	0.019276
mmu01210	2-Oxocarboxylic acid metabolism	9	0	9	144	0.002601	0.019276

mmu00360	Phenylalanine metabolism	5	0	5	49	0.003102	0.021711
mmu04976	Bile secretion	4	3	7	97	0.003559	0.023604
mmu00230	Purine metabolism	6	1	7	101	0.004459	0.028093
mmu00400	Phenylalanine, tyrosine and tryptophan biosynthesis	3	1	4	35	0.005421	0.032526
mmu00020	TCA cycle	3	0	3	20	0.007487	0.042881
mmu00220	Arginine biosynthesis	3	0	3	23	0.011125	0.058408
mmu00290	Valine, leucine and isoleucine biosynthesis	3	0	3	23	0.011125	0.058408
mmu04922	Glucagon signaling pathway	3	0	3	26	0.015625	0.076905
mmu01240	Biosynthesis of cofactors	11	2	13	328	0.015869	0.076905
mmu04540	Gap junction	2	0	2	11	0.020475	0.092136
mmu04917	Prolactin signaling pathway	2	0	2	11	0.020475	0.092136
mmu04971	Gastric acid secretion	2	0	2	14	0.03255	0.141422
mmu01100	Metabolic pathways	53	18	71	3063	0.037797	0.158748
mmu00190	Oxidative phosphorylation	2	0	2	16	0.041799	0.159201
mmu04270	Vascular smooth muscle contraction	1	1	2	16	0.041799	0.159201
mmu00630	Glyoxylate and dicarboxylate metabolism	4	0	4	64	0.04197	0.159201
mmu04977	Vitamin digestion and absorption	3	0	3	39	0.045385	0.159201
mmu04924	Renin secretion	2	0	2	17	0.046749	0.159201

mmu04964	Proximal tubule bicarbonate reclamation	2	0	2	17	0.046749	0.159201
mmu04970	Salivary secretion	2	0	2	17	0.046749	0.159201
mmu00270	Cysteine and methionine metabolism	3	1	4	67	0.048354	0.160331
mmu00330	Arginine and proline metabolism	4	0	4	69	0.052893	0.170886
mmu04726	Serotonergic synapse	1	2	3	42	0.054575	0.171912
mmu05224	Breast cancer	1	0	1	3	0.060582	0.186179
mmu00260	Glycine, serine and threonine metabolism	3	0	3	48	0.07538	0.226139
mmu04142	Lysosome	1	0	1	4	0.079958	0.228972
mmu04150	mTOR signaling pathway	1	0	1	4	0.079958	0.228972
mmu00430	Taurine and hypotaurine metabolism	2	0	2	24	0.086535	0.237032
mmu00740	Riboflavin metabolism	2	0	2	24	0.086535	0.237032
mmu01522	Endocrine resistance	1	0	1	5	0.098939	0.240551
mmu03320	PPAR signaling pathway	0	1	1	5	0.098939	0.240551
mmu04068	FoxO signaling pathway	1	0	1	5	0.098939	0.240551
mmu04810	Regulation of actin cytoskeleton	1	0	1	5	0.098939	0.240551
mmu05310	Asthma	1	0	1	5	0.098939	0.240551
mmu05012	Parkinson disease	2	0	2	26	0.099275	0.240551
mmu00450	Selenocompound metabolism	0	2	2	27	0.105831	0.251264

mmu00310	Lysine degradation	3	0	3	56	0.107685	0.251264
mmu05208	Chemical carcinogenesis - reactive oxygen species	2	1	3	57	0.112053	0.253129
mmu00591	Linoleic acid metabolism	0	2	2	28	0.112502	0.253129
mmu00750	Vitamin B6 metabolism	2	0	2	29	0.119279	0.254732
mmu00780	Biotin metabolism	2	0	2	29	0.119279	0.254732
mmu05207	Chemical carcinogenesis - receptor activation	2	0	2	29	0.119279	0.254732
mmu00770	Pantothenate and CoA biosynthesis	2	0	2	30	0.126156	0.264928
mmu05030	Cocaine addiction	1	0	1	7	0.135747	0.275873
mmu05033	Nicotine addiction	1	0	1	7	0.135747	0.275873
mmu00410	beta-Alanine metabolism	2	0	2	32	0.140182	0.275983
mmu05022	Pathways of neurodegeneration - multiple diseases	2	0	2	32	0.140182	0.275983
mmu00920	Sulfur metabolism	2	0	2	33	0.147317	0.276461
mmu04724	Glutamatergic synapse	1	0	1	8	0.15359	0.276461
mmu04915	Estrogen signaling pathway	1	0	1	8	0.15359	0.276461
mmu04961	Endocrine and other factor-regulated calcium reabsorption	1	0	1	8	0.15359	0.276461
mmu05032	Morphine addiction	1	0	1	8	0.15359	0.276461
mmu05143	African trypanosomiasis	1	0	1	8	0.15359	0.276461
mmu04750	Inflammatory mediator regulation of TRP channels	1	1	2	35	0.161804	0.287144

mmu04929	GnRH secretion	1	0	1	9	0.171068	0.295267
mmu05031	Amphetamine addiction	1	0	1	9	0.171068	0.295267
mmu00480	Glutathione metabolism	2	0	2	38	0.183983	0.311997
mmu04261	Adrenergic signaling in cardiomyocytes	1	0	1	10	0.188189	0.311997
mmu05034	Alcoholism	1	0	1	10	0.188189	0.311997
mmu05415	Diabetic cardiomyopathy	1	1	2	39	0.191475	0.313323
mmu01040	Biosynthesis of unsaturated fatty acids	0	3	3	74	0.195145	0.315234
mmu04664	Fc epsilon RI signaling pathway	1	0	1	11	0.20496	0.318827
mmu05215	Prostate cancer	1	0	1	11	0.20496	0.318827
mmu05231	Choline metabolism in cancer	1	0	1	11	0.20496	0.318827
mmu01200	Carbon metabolism	4	0	4	114	0.207531	0.318889
mmu00280	Valine, leucine and isoleucine degradation	2	0	2	42	0.214176	0.321509
mmu04725	Cholinergic synapse	1	0	1	12	0.221389	0.321509
mmu04728	Dopaminergic synapse	1	0	1	12	0.221389	0.321509
mmu04911	Insulin secretion	1	0	1	12	0.221389	0.321509
mmu00590	Arachidonic acid metabolism	0	3	3	79	0.221994	0.321509
mmu00785	Lipoic acid metabolism	2	0	2	44	0.229453	0.328535
mmu00380	Tryptophan metabolism	3	0	3	83	0.243989	0.343107

mmu00650	Butanoate metabolism	2	0	2	47	0.252498	0.343107
mmu04611	Platelet activation	1	0	1	14	0.253246	0.343107
mmu04923	Regulation of lipolysis in adipocytes	1	0	1	14	0.253246	0.343107
mmu05014	Amyotrophic lateral sclerosis	1	0	1	14	0.253246	0.343107
mmu04066	HIF-1 signaling pathway	1	0	1	15	0.268688	0.352652
mmu04071	Sphingolipid signaling pathway	0	1	1	15	0.268688	0.352652
mmu04972	Pancreatic secretion	1	0	1	15	0.268688	0.352652
mmu01523	Antifolate resistance	1	0	1	17	0.29863	0.387911
mmu00564	Glycerophospholipid metabolism	2	0	2	56	0.321667	0.413572
mmu00910	Nitrogen metabolism	1	0	1	19	0.32736	0.414905
mmu00053	Ascorbate and aldarate metabolism	1	1	2	57	0.32929	0.414905
mmu00040	Pentose and glucuronate interconversions	1	1	2	58	0.33689	0.420278
mmu00232	Caffeine metabolism	1	0	1	22	0.368286	0.450525
mmu04152	AMPK signaling pathway	1	0	1	22	0.368286	0.450525
mmu00240	Pyrimidine metabolism	2	0	2	64	0.3819	0.462686
mmu04913	Ovarian steroidogenesis	1	0	1	24	0.394192	0.473031
mmu05200	Pathways in cancer	1	0	1	31	0.476861	0.566834
mmu00620	Pyruvate metabolism	1	0	1	32	0.487721	0.574326

mmu00600	Sphingolipid metabolism	0	1	1	35	0.518983	0.60548
mmu00030	Pentose phosphate pathway	1	0	1	36	0.528978	0.61148
mmu00640	Propanoate metabolism	1	0	1	41	0.575957	0.659732
mmu00592	alpha-Linolenic acid metabolism	0	1	1	44	0.601887	0.683223
mmu00140	Steroid hormone biosynthesis	2	0	2	99	0.610895	0.686151
mmu00052	Galactose metabolism	0	1	1	46	0.618297	0.686151
mmu00120	Primary bile acid biosynthesis	1	0	1	47	0.626249	0.686151
mmu00562	Inositol phosphate metabolism	0	1	1	47	0.626249	0.686151
mmu00071	Fatty acid degradation	1	0	1	50	0.649136	0.705096
mmu00983	Drug metabolism - other enzymes	1	0	1	52	0.663618	0.714665
mmu00051	Fructose and mannose metabolism	0	1	1	55	0.68424	0.724489
mmu00760	Nicotinate and nicotinamide metabolism	1	0	1	55	0.68424	0.724489
mmu00100	Steroid biosynthesis	1	0	1	57	0.697288	0.732153
mmu00520	Amino sugar and nucleotide sugar metabolism	0	2	2	118	0.705879	0.735047
mmu00130	Ubiquinone and other terpenoid-quinone biosynthesis	0	1	1	71	0.774841	0.800245
mmu00524	Neomycin, kanamycin and gentamicin biosynthesis	1	0	1	81	0.817857	0.837804
mmu01250	Biosynthesis of nucleotide sugars	0	2	2	200	0.923919	0.931431
mmu01212	Fatty acid metabolism	1	0	1	122	0.924039	0.931431

mmu00860	Porphyrin metabolism	1	0	1	148	0.956571	0.956571
