# Supplementary material for: Maternal Cadmium Exposure Impairs Lactational Performance and Milk Quality in Mice
Source: Biology (Basel). 2026 May 9;15(10):754. doi: 10.3390/biology15100754 (PMC13203474; doi:10.3390/biology15100754)
Supplement: Supplementary file 1 [file biology-15-00754-s001.zip › Table S3.pdf]

Table S3. Identified differential genes from transcriptomic analyses of mammary tissue

PathwayID	Pathway	Up	Down	DEG	Total	P	AdjustP
mmu04640	Hematopoietic cell lineage	28	1	29	94	7.61E-22	2.11E-19
mmu05340	Primary immunodeficiency	18	0	18	36	1.86E-18	2.58E-16
mmu04514	Cell adhesion molecules	31	0	31	163	1.39E-16	1.28E-14
mmu04672	Intestinal immune network for IgA production	17	0	17	43	2.41E-15	1.67E-13
mmu04659	Th17 cell differentiation	24	0	24	105	5.83E-15	3.23E-13
mmu04658	Th1 and Th2 cell differentiation	22	0	22	88	1.11E-14	5.12E-13
mmu05169	Epstein-Barr virus infection	29	4	33	224	3.42E-14	1.35E-12
mmu04612	Antigen processing and presentation	18	2	20	83	4.00E-13	1.38E-11
mmu04662	B cell receptor signaling pathway	19	0	19	77	9.94E-13	3.06E-11
mmu05416	Viral myocarditis	19	0	19	81	2.66E-12	7.35E-11
mmu05330	Allograft rejection	16	0	16	56	5.59E-12	1.41E-10
mmu04660	T cell receptor signaling pathway	20	0	20	103	2.90E-11	6.70E-10
mmu05310	Asthma	11	0	11	25	5.73E-11	1.22E-09
mmu05166	Human T-cell leukemia virus 1 infection	27	2	29	243	2.68E-10	5.31E-09
mmu05320	Autoimmune thyroid disease	16	0	16	72	3.55E-10	6.56E-09
mmu05332	Graft-versus-host disease	14	0	14	56	8.72E-10	1.51E-08
mmu04940	Type I diabetes mellitus	14	0	14	63	4.63E-09	7.54E-08

mmu04145	Phagosome	19	3	22	173	1.34E-08	2.06E-07
mmu04064	NF-kappa B signaling pathway	17	0	17	105	1.69E-08	2.46E-07
mmu05152	Tuberculosis	20	2	22	179	2.52E-08	3.49E-07
mmu05323	Rheumatoid arthritis	15	0	15	87	5.03E-08	6.64E-07
mmu04060	Cytokine-cytokine receptor interaction	27	1	28	289	5.96E-08	7.50E-07
mmu05140	Leishmaniasis	13	0	13	70	1.62E-07	1.95E-06
mmu05321	Inflammatory bowel disease	12	0	12	62	3.04E-07	3.51E-06
mmu04062	Chemokine signaling pathway	21	0	21	190	3.33E-07	3.69E-06
mmu05145	Toxoplasmosis	15	0	15	109	1.06E-06	1.13E-05
mmu04650	Natural killer cell mediated cytotoxicity	17	0	17	141	1.35E-06	1.39E-05
mmu05235	PD-L1 expression and PD-1 checkpoint pathway in cancer	12	0	12	88	1.42E-05	0.000140613
mmu05150	Staphylococcus aureus infection	13	1	14	120	1.73E-05	0.000165014
mmu05164	Influenza A	15	2	17	173	2.19E-05	0.000201159
mmu04061	Viral protein interaction with cytokine and cytokine receptor	11	1	12	92	2.25E-05	0.000201159
mmu05135	Yersinia infection	14	0	14	135	6.52E-05	0.000564312
mmu05322	Systemic lupus erythematosus	13	1	14	143	0.000122	0.001024345

mmu05170	Human immunodeficiency virus 1 infection	16	2	18	233	0.000291	0.002370997
mmu05168	Herpes simplex virus 1 infection	23	4	27	441	0.000464	0.003672839
mmu04670	Leukocyte transendothelial migration	11	0	11	114	0.000733	0.005642269
mmu05162	Measles	9	3	12	146	0.001758	0.013161732
mmu05132	Salmonella infection	16	1	17	251	0.001854	0.013516827
mmu04666	Fc gamma R-mediated phagocytosis	9	0	9	93	0.002156	0.01531431
mmu04664	Fc epsilon RI signaling pathway	7	0	7	66	0.003965	0.027460036
mmu04621	NOD-like receptor signaling pathway	12	2	14	205	0.004258	0.028456887
mmu05142	Chagas disease	8	1	9	103	0.004315	0.028456887
mmu05144	Malaria	5	1	6	55	0.006537	0.042109999
mmu04610	Complement and coagulation cascades	5	3	8	91	0.006734	0.042390506
mmu04810	Regulation of actin cytoskeleton	14	0	14	219	0.00756	0.046533251
mmu04620	Toll-like receptor signaling pathway	7	1	8	99	0.011006	0.066273485
mmu04380	Osteoclast differentiation	9	0	9	124	0.014014	0.082592083
mmu04015	Rap1 signaling pathway	13	0	13	214	0.014687	0.084758378
mmu05417	Lipid and atherosclerosis	12	1	13	216	0.015761	0.08909859
mmu05163	Human cytomegalovirus infection	13	1	14	248	0.020681	0.114574745
mmu04725	Cholinergic synapse	8	0	8	112	0.021799	0.118396817

mmu05167	Kaposi sarcoma-associated herpesvirus infection	12	0	12	217	0.035384	0.18848843
mmu04510	Focal adhesion	9	2	11	200	0.044302	0.228420777
mmu04921	Oxytocin signaling pathway	9	0	9	152	0.04453	0.228420777
mmu05146	Amoebiasis	5	2	7	107	0.046382	0.233597444
mmu04928	Parathyroid hormone synthesis, secretion and action	5	2	7	108	0.048356	0.23918907
mmu04625	C-type lectin receptor signaling pathway	5	2	7	112	0.056781	0.275937259
mmu04371	Apelin signaling pathway	7	1	8	136	0.058126	0.277600804
mmu05032	Morphine addiction	6	0	6	91	0.060852	0.285696027
mmu00562	Inositol phosphate metabolism	5	0	5	72	0.070036	0.323335147
mmu04630	JAK-STAT signaling pathway	8	1	9	167	0.072008	0.326989213
mmu04022	cGMP-PKG signaling pathway	9	0	9	170	0.07854	0.350897946
mmu04713	Circadian entrainment	5	1	6	98	0.080831	0.354103085
mmu04151	PI3K-Akt signaling pathway	13	3	16	356	0.081814	0.354103085
mmu04611	Platelet activation	7	0	7	124	0.087234	0.368042495
mmu05412	Arrhythmogenic right ventricular cardiomyopathy	5	0	5	77	0.087692	0.368042495
mmu04613	Neutrophil extracellular trap formation	10	0	10	201	0.090923	0.37590647
mmu04370	VEGF signaling pathway	4	0	4	58	0.102237	0.407013823
mmu04960	Aldosterone-regulated sodium reabsorption	2	1	3	37	0.103968	0.407013823

mmu05143	African trypanosomiasis	3	0	3	37	0.103968	0.407013823
mmu00120	Primary bile acid biosynthesis	2	0	2	18	0.104325	0.407013823
mmu04970	Salivary secretion	5	0	5	83	0.111639	0.425806006
mmu05134	Legionellosis	4	0	4	60	0.112216	0.425806006
mmu05202	Transcriptional misregulation in cancer	10	0	10	212	0.117947	0.441502706
mmu04020	Calcium signaling pathway	10	1	11	240	0.120943	0.446682149
mmu04929	GnRH secretion	3	1	4	63	0.127956	0.459586906
mmu04972	Pancreatic secretion	6	0	6	112	0.130385	0.459586906
mmu04512	ECM-receptor interaction	3	2	5	88	0.13375	0.459586906
mmu05210	Colorectal cancer	5	0	5	88	0.13375	0.459586906
mmu04724	Glutamatergic synapse	5	1	6	113	0.134386	0.459586906
mmu00770	Pantothenate and CoA biosynthesis	2	0	2	21	0.135	0.459586906
mmu04936	Alcoholic liver disease	6	1	7	139	0.136051	0.459586906
mmu04727	GABAergic synapse	5	0	5	89	0.138393	0.461865462
mmu04071	Sphingolipid signaling pathway	6	0	6	120	0.163967	0.540700907
mmu04973	Carbohydrate digestion and absorption	3	0	3	46	0.167198	0.540856615
mmu05221	Acute myeloid leukemia	4	0	4	70	0.167919	0.540856615
mmu00232	Caffeine metabolism	0	1	1	6	0.170864	0.541421327

mmu04070	Phosphatidylinositol signaling system	5	0	5	96	0.17278	0.541421327
mmu04520	Adherens junction	4	0	4	71	0.173958	0.541421327
mmu00592	alpha-Linolenic acid metabolism	2	0	2	25	0.178431	0.549171141
mmu04918	Thyroid hormone synthesis	3	1	4	73	0.186256	0.563328607
mmu05171	Coronavirus disease - COVID-19	4	6	10	235	0.187098	0.563328607
mmu04750	Inflammatory mediator regulation of TRP channels	5	1	6	126	0.191333	0.569882951
mmu04916	Melanogenesis	5	0	5	100	0.193773	0.571011684
mmu04971	Gastric acid secretion	4	0	4	75	0.198825	0.573626086
mmu04933	AGE-RAGE signaling pathway in diabetic complications	5	0	5	101	0.199157	0.573626086
mmu00650	Butanoate metabolism	2	0	2	27	0.200873	0.573626086
mmu05133	Pertussis	4	0	4	76	0.205205	0.576866513
mmu00380	Tryptophan metabolism	3	0	3	51	0.206173	0.576866513
mmu00740	Riboflavin metabolism	1	0	1	8	0.221088	0.612414768
mmu04210	Apoptosis	5	1	6	136	0.240399	0.659312224
mmu00280	Valine, leucine and isoleucine degradation	3	0	3	56	0.246942	0.664107355
mmu04923	Regulation of lipolysis in adipocytes	3	0	3	56	0.246942	0.664107355
mmu04310	Wnt signaling pathway	7	0	7	167	0.25412	0.674321125

mmu04723	Retrograde endocannabinoid signaling	5	1	6	139	0.255816	0.674321125
mmu04215	Apoptosis - multiple species	2	0	2	32	0.258043	0.674321125
mmu05213	Endometrial cancer	3	0	3	58	0.263596	0.679123428
mmu04911	Insulin secretion	4	0	4	85	0.264941	0.679123428
mmu04066	HIF-1 signaling pathway	5	0	5	113	0.267236	0.679123428
mmu00590	Arachidonic acid metabolism	3	1	4	86	0.271783	0.684400027
mmu04961	Endocrine and other factor-regulated calcium reabsorption	2	1	3	60	0.280384	0.695105602
mmu01040	Biosynthesis of unsaturated fatty acids	1	1	2	34	0.281054	0.695105602
mmu03320	PPAR signaling pathway	3	1	4	89	0.292482	0.716968
mmu04261	Adrenergic signaling in cardiomyocytes	6	0	6	147	0.298118	0.724375093
mmu04919	Thyroid hormone signaling pathway	4	1	5	119	0.303064	0.729988067
mmu04072	Phospholipase D signaling pathway	6	0	6	149	0.308911	0.73765739
mmu04914	Progesterone-mediated oocyte maturation	3	1	4	92	0.313381	0.740476103
mmu05216	Thyroid cancer	2	0	2	37	0.315437	0.740476103
mmu04360	Axon guidance	5	2	7	181	0.322046	0.749635897
mmu05414	Dilated cardiomyopathy	4	0	4	94	0.327391	0.755727671
mmu05033	Nicotine addiction	1	1	2	40	0.349443	0.799965021

mmu00533	Glycosaminoglycan biosynthesis - keratan sulfate	1	0	1	14	0.354291	0.80060967
mmu05231	Choline metabolism in cancer	4	0	4	98	0.355505	0.80060967
mmu05215	Prostate cancer	4	0	4	99	0.36254	0.809866809
mmu04142	Lysosome	5	0	5	130	0.370259	0.820493434
mmu00604	Glycosphingolipid biosynthesis - ganglio series	1	0	1	15	0.37417	0.8209774
mmu04068	FoxO signaling pathway	2	3	5	131	0.376405	0.8209774
mmu04115	p53 signaling pathway	2	1	3	72	0.381873	0.82211866
mmu04975	Fat digestion and absorption	1	1	2	43	0.382864	0.82211866
mmu00230	Purine metabolism	4	1	5	133	0.388694	0.828216596
mmu00603	Glycosphingolipid biosynthesis - globo and isoglobo series	1	0	1	16	0.39344	0.831930938
mmu04924	Renin secretion	3	0	3	75	0.406936	0.851983517
mmu00910	Nitrogen metabolism	0	1	1	17	0.412119	0.851983517
mmu05100	Bacterial invasion of epithelial cells	3	0	3	76	0.415227	0.851983517
mmu05212	Pancreatic cancer	3	0	3	76	0.415227	0.851983517
mmu04014	Ras signaling pathway	8	0	8	232	0.42129	0.85806837
mmu00511	Other glycan degradation	1	0	1	18	0.430224	0.869869792
mmu00565	Ether lipid metabolism	2	0	2	48	0.436827	0.870304004

mmu04930	Type II diabetes mellitus	2	0	2	48	0.436827	0.870304004
mmu01521	EGFR tyrosine kinase inhibitor resistance	3	0	3	79	0.439865	0.870304004
mmu04979	Cholesterol metabolism	1	1	2	49	0.447316	0.870743158
mmu01524	Platinum drug resistance	3	0	3	80	0.447991	0.870743158
mmu04218	Cellular senescence	5	1	6	176	0.457332	0.870743158
mmu00591	Linoleic acid metabolism	2	0	2	50	0.457696	0.870743158
mmu00100	Steroid biosynthesis	0	1	1	20	0.464786	0.870743158
mmu00520	Amino sugar and nucleotide sugar metabolism	2	0	2	51	0.467962	0.870743158
mmu00071	Fatty acid degradation	2	0	2	52	0.478112	0.870743158
mmu02010	ABC transporters	2	0	2	52	0.478112	0.870743158
mmu05204	Chemical carcinogenesis - DNA adducts	2	1	3	84	0.480001	0.870743158
mmu04935	Growth hormone synthesis, secretion and action	4	0	4	116	0.480105	0.870743158
mmu00531	Glycosaminoglycan degradation	1	0	1	21	0.481275	0.870743158
mmu00532	Glycosaminoglycan biosynthesis - chondroitin sulfate / dermatan sulfate	1	0	1	21	0.481275	0.870743158
mmu04964	Proximal tubule bicarbonate reclamation	1	0	1	21	0.481275	0.870743158
mmu04978	Mineral absorption	1	1	2	53	0.488144	0.870743158
mmu04540	Gap junction	3	0	3	86	0.49568	0.870743158

mmu03430	Mismatch repair	0	1	1	22	0.497258	0.870743158
mmu04024	cAMP signaling pathway	7	0	7	216	0.498047	0.870743158
mmu00900	Terpenoid backbone biosynthesis	1	0	1	23	0.512751	0.870743158
mmu04722	Neurotrophin signaling pathway	4	0	4	121	0.51321	0.870743158
mmu05165	Human papillomavirus infection	8	3	11	352	0.521974	0.870743158
mmu04912	GnRH signaling pathway	3	0	3	90	0.52631	0.870743158
mmu04742	Taste transduction	2	1	3	91	0.533807	0.870743158
mmu05410	Hypertrophic cardiomyopathy	3	0	3	91	0.533807	0.870743158
mmu00240	Pyrimidine metabolism	1	1	2	58	0.536451	0.870743158
mmu05415	Diabetic cardiomyopathy	6	0	6	191	0.537151	0.870743158
mmu00983	Drug metabolism - other enzymes	1	2	3	92	0.541237	0.870743158
mmu01522	Endocrine resistance	3	0	3	93	0.548599	0.870743158
mmu04730	Long-term depression	2	0	2	60	0.554881	0.870743158
mmu00340	Histidine metabolism	1	0	1	26	0.556432	0.870743158
mmu00601	Glycosphingolipid biosynthesis - lacto and neolacto series	1	0	1	26	0.556432	0.870743158
mmu04934	Cushing syndrome	4	1	5	162	0.560199	0.870743158
mmu05160	Hepatitis C	3	2	5	162	0.560199	0.870743158

mmu04926	Relaxin signaling pathway	4	0	4	129	0.564065	0.870743158
mmu05161	Hepatitis B	5	0	5	163	0.565718	0.870743158
mmu03060	Protein export	0	1	1	27	0.570107	0.870743158
mmu04744	Phototransduction	1	0	1	27	0.570107	0.870743158
mmu04950	Maturity onset diabetes of the young	1	0	1	27	0.570107	0.870743158
mmu04144	Endocytosis	8	0	8	265	0.572018	0.870743158
mmu00310	Lysine degradation	2	0	2	62	0.572787	0.870743158
mmu00561	Glycerolipid metabolism	1	1	2	62	0.572787	0.870743158
mmu04213	Longevity regulating pathway - multiple species	2	0	2	62	0.572787	0.870743158
mmu04726	Serotonergic synapse	4	0	4	131	0.576322	0.870743158
mmu04728	Dopaminergic synapse	3	1	4	131	0.576322	0.870743158
mmu04623	Cytosolic DNA-sensing pathway	2	0	2	63	0.581543	0.870743158
mmu05217	Basal cell carcinoma	2	0	2	63	0.581543	0.870743158
mmu04976	Bile secretion	3	0	3	99	0.59128	0.874321871
mmu04915	Estrogen signaling pathway	4	0	4	134	0.594338	0.874321871
mmu01523	Antifolate resistance	1	0	1	29	0.59621	0.874321871
mmu04080	Neuroactive ligand-receptor interaction	6	5	11	372	0.596967	0.874321871
mmu04720	Long-term potentiation	2	0	2	66	0.607017	0.874321871

mmu04710	Circadian rhythm	0	1	1	30	0.608664	0.874321871
mmu04925	Aldosterone synthesis and secretion	3	0	3	102	0.611625	0.874321871
mmu00053	Ascorbate and aldarate metabolism	1	0	1	31	0.620734	0.874321871
mmu04922	Glucagon signaling pathway	2	1	3	104	0.624808	0.874321871
mmu04140	Autophagy - animal	3	1	4	140	0.628982	0.874321871
mmu00410	beta-Alanine metabolism	1	0	1	32	0.632434	0.874321871
mmu00512	Mucin type O-glycan biosynthesis	1	0	1	32	0.632434	0.874321871
mmu04130	SNARE interactions in vesicular transport	1	0	1	32	0.632434	0.874321871
mmu04136	Autophagy - other	0	1	1	32	0.632434	0.874321871
mmu05017	Spinocerebellar ataxia	4	0	4	141	0.63457	0.874321871
mmu00500	Starch and sucrose metabolism	1	0	1	33	0.643774	0.874321871
mmu03410	Base excision repair	1	0	1	33	0.643774	0.874321871
mmu04270	Vascular smooth muscle contraction	4	0	4	143	0.645582	0.874321871
mmu00982	Drug metabolism - cytochrome P450	2	0	2	71	0.646833	0.874321871
mmu00480	Glutathione metabolism	2	0	2	72	0.654403	0.874321871
mmu04927	Cortisol synthesis and secretion	2	0	2	72	0.654403	0.874321871
mmu05223	Non-small cell lung cancer	2	0	2	72	0.654403	0.874321871
mmu00980	Metabolism of xenobiotics by cytochrome P450	2	0	2	73	0.661844	0.874321871

mmu05020	Prion disease	5	2	7	253	0.665559	0.874321871
mmu05224	Breast cancer	4	0	4	147	0.666947	0.874321871
mmu04917	Prolactin signaling pathway	2	0	2	74	0.669156	0.874321871
mmu05214	Glioma	2	0	2	74	0.669156	0.874321871
mmu00051	Fructose and mannose metabolism	1	0	1	36	0.675746	0.876686721
mmu05226	Gastric cancer	4	0	4	149	0.677296	0.876686721
mmu04010	MAPK signaling pathway	8	0	8	294	0.688194	0.886649781
mmu00260	Glycine, serine and threonine metabolism	1	0	1	40	0.71397	0.907200937
mmu00513	Various types of N-glycan biosynthesis	1	0	1	40	0.71397	0.907200937
mmu04216	Ferroptosis	1	0	1	40	0.71397	0.907200937
mmu00760	Nicotinate and nicotinamide metabolism	1	0	1	41	0.722803	0.914230577
mmu04152	AMPK signaling pathway	2	1	3	123	0.734563	0.921761632
mmu04012	ErbB signaling pathway	2	0	2	84	0.735413	0.921761632
mmu00514	Other types of O-glycan biosynthesis	1	0	1	43	0.739662	0.9229115
mmu00970	Aminoacyl-tRNA biosynthesis	1	0	1	44	0.747704	0.92461635
mmu04962	Vasopressin-regulated water reabsorption	1	0	1	44	0.747704	0.92461635
mmu00620	Pyruvate metabolism	1	0	1	45	0.755499	0.93010314
mmu04211	Longevity regulating pathway	2	0	2	90	0.769506	0.943155788

mmu05207	Chemical carcinogenesis - receptor activation	5	0	5	209	0.775469	0.946277705
mmu05225	Hepatocellular carcinoma	4	0	4	173	0.784059	0.949420015
mmu05222	Small cell lung cancer	2	0	2	93	0.785079	0.949420015
mmu05200	Pathways in cancer	14	0	14	542	0.788327	0.949420015
mmu04350	TGF-beta signaling pathway	2	0	2	95	0.794942	0.953241687
mmu00564	Glycerophospholipid metabolism	2	0	2	98	0.808988	0.961475227
mmu04550	Signaling pathways regulating pluripotency of stem cells	3	0	3	140	0.80987	0.961475227
mmu05203	Viral carcinogenesis	5	0	5	221	0.815172	0.961475227
mmu00330	Arginine and proline metabolism	1	0	1	54	0.815692	0.961475227
mmu05418	Fluid shear stress and atherosclerosis	3	0	3	145	0.828286	0.968813084
mmu05022	Pathways of neurodegeneration - multiple diseases	9	2	11	456	0.836653	0.968813084
mmu04340	Hedgehog signaling pathway	1	0	1	58	0.837463	0.968813084
mmu04932	Non-alcoholic fatty liver disease	3	0	3	150	0.845158	0.968813084
mmu04974	Protein digestion and absorption	1	1	2	107	0.846126	0.968813084
mmu04330	Notch signaling pathway	1	0	1	60	0.847368	0.968813084
mmu04931	Insulin resistance	2	0	2	109	0.853437	0.968813084
mmu04913	Ovarian steroidogenesis	1	0	1	63	0.861108	0.968813084

mmu04390	Hippo signaling pathway	3	0	3	156	0.863498	0.968813084
mmu04150	mTOR signaling pathway	3	0	3	157	0.866363	0.968813084
mmu04668	TNF signaling pathway	2	0	2	113	0.867119	0.968813084
mmu05206	MicroRNAs in cancer	3	0	3	161	0.8773	0.968813084
mmu00010	Glycolysis / Gluconeogenesis	1	0	1	67	0.877531	0.968813084
mmu04137	Mitophagy - animal	0	1	1	68	0.881325	0.968813084
mmu05031	Amphetamine addiction	1	0	1	68	0.881325	0.968813084
mmu05211	Renal cell carcinoma	1	0	1	68	0.881325	0.968813084
mmu05230	Central carbon metabolism in cancer	1	0	1	69	0.885002	0.968813084
mmu04114	Oocyte meiosis	1	1	2	120	0.888281	0.968813084
mmu04622	RIG-I-like receptor signaling pathway	0	1	1	70	0.888565	0.968813084
mmu04920	Adipocytokine signaling pathway	1	0	1	71	0.892018	0.968813084
mmu05218	Melanoma	1	0	1	72	0.895365	0.968813084
mmu04141	Protein processing in endoplasmic reticulum	1	2	3	170	0.89906	0.969026021
mmu04217	Necroptosis	3	0	3	174	0.90757	0.970150532
mmu05220	Chronic myeloid leukemia	1	0	1	76	0.90775	0.970150532
mmu04721	Synaptic vesicle cycle	0	1	1	77	0.910611	0.970150532
mmu03018	RNA degradation	1	0	1	80	0.918673	0.974990627

mmu04260	Cardiac muscle contraction	1	0	1	82	0.923642	0.976522214
mmu04910	Insulin signaling pathway	2	0	2	138	0.92921	0.978617771
mmu04146	Peroxisome	1	0	1	86	0.93269	0.978617771
mmu05010	Alzheimer disease	6	1	7	366	0.938066	0.979768676
mmu00140	Steroid hormone biosynthesis	1	0	1	91	0.942512	0.979768676
mmu05016	Huntington disease	4	1	5	287	0.944398	0.979768676
mmu05205	Proteoglycans in cancer	3	0	3	201	0.949947	0.981848616
mmu05014	Amyotrophic lateral sclerosis	4	2	6	352	0.963277	0.989156334
mmu04530	Tight junction	1	1	2	164	0.96416	0.989156334
mmu04110	Cell cycle	0	1	1	125	0.980382	0.998693659
mmu03010	Ribosome	0	1	1	130	0.983257	0.998693659
mmu05012	Parkinson disease	1	2	3	249	0.984272	0.998693659
mmu05208	Chemical carcinogenesis - reactive oxygen species	2	0	2	207	0.98886	0.999686719
mmu05034	Alcoholism	1	0	1	199	0.998139	1
mmu04714	Thermogenesis	1	0	1	215	0.998885	1
mmu04740	Olfactory transduction	2	2	4	1151	1	1
