# Supplementary figures and images for: Maternal Cadmium Exposure Impairs Lactational Performance and Milk Quality in Mice
Source: Biology (Basel). 2026 May 9;15(10):754. doi: 10.3390/biology15100754 (PMC13203474; doi:10.3390/biology15100754)

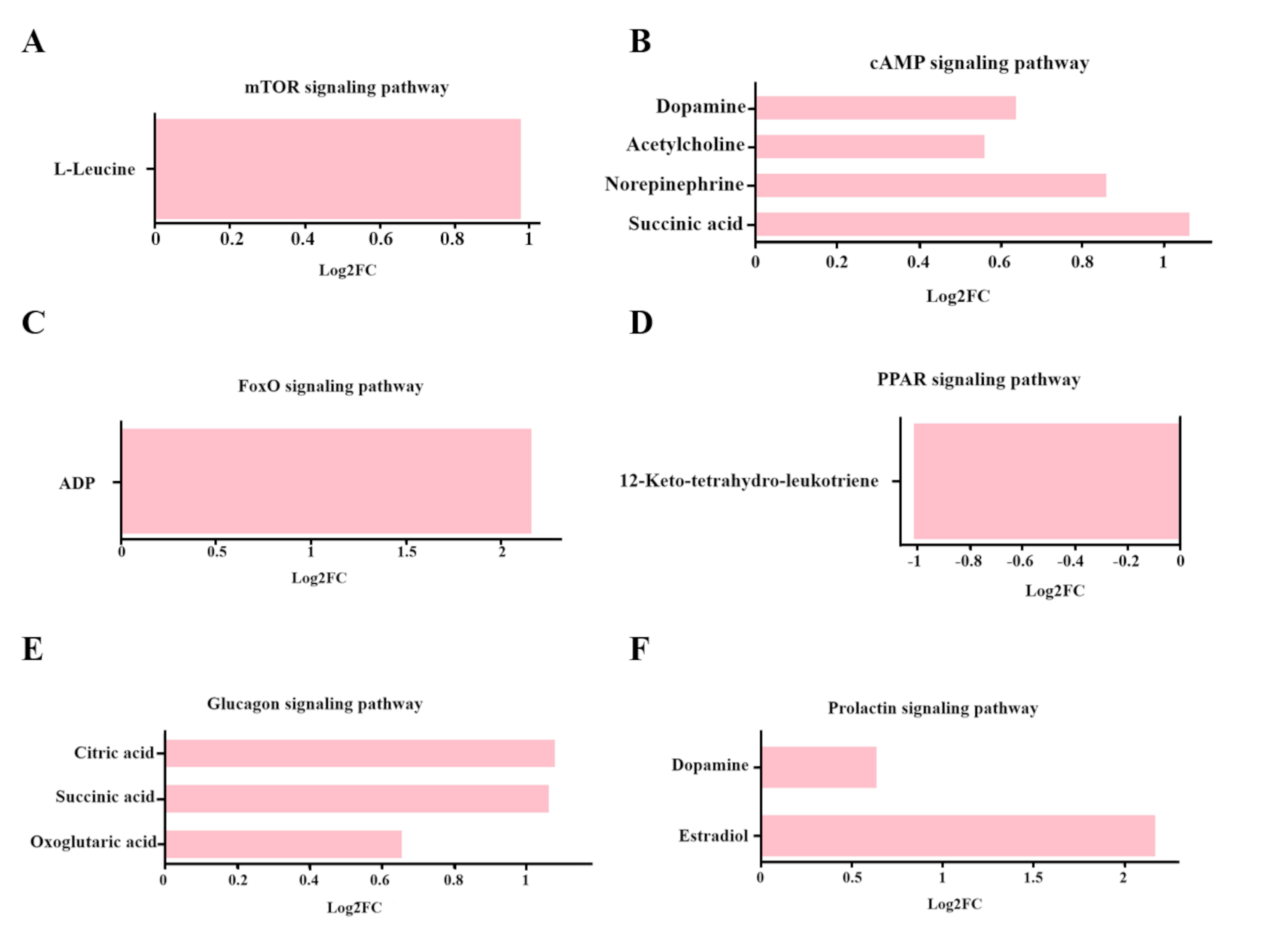

Supplement: Supplementary file 1 [file biology-15-00754-s001.zip › Figure.S1.tiff]
